# The impact of COVID-19 and social distancing on people with Parkinson’s disease: a survey study

**DOI:** 10.1038/s41531-020-00153-8

**Published:** 2021-01-21

**Authors:** Megan P. Feeney, Yaqian Xu, Matthew Surface, Hiral Shah, Nora Vanegas-Arroyave, Amanda K. Chan, Elizabeth Delaney, Serge Przedborski, James C. Beck, Roy N. Alcalay

**Affiliations:** 1 Parkinson’s Foundation, New York, NY USA; 2 Department of Psychiatry, Columbia University Irving Medical Center, New York, NY USA; 3 Department of Neurology, Columbia University Irving Medical Center, New York, NY USA; 4 Department of Neurology, Parkinson’s Disease Center and Movement Disorder Clinic, Baylor College of Medicine, Houston, TX USA; 5 Departments of Neurology, Pathology & Cell Biology and Neuroscience, Columbia University Irving Medical Center, New York, NY USA; 6 Department of Neuroscience and Physiology, NYU Grossman School of Medicine, New York, NY USA

**Keywords:** Parkinson's disease, Health policy, Parkinson's disease

## Abstract

As the COVID-19 pandemic continues to affect the international community, very little is known about its impact on the health and day-to-day activities of people with Parkinson’s disease (PwPD). To better understand the emotional and behavioral consequences of the public health policies implemented to mitigate the spread of SARS-CoV-2 in PwPD, and to explore the factors contributing to accessing alternative health care mechanisms, such as telehealth, we administered an anonymous knowledge, attitude, and practice survey to PwPD and care partners, via the mailing lists of the Parkinson’s Foundation and Columbia University Parkinson’s Disease Center of Excellence with an average response rate of 19.3%. Sufficient information was provided by 1,342 PwPD to be included in the final analysis. Approximately half of respondents reported a negative change in PD symptoms, with 45–66% reporting mood disturbances. Telehealth use increased from 9.7% prior to the pandemic to 63.5% during the pandemic. Higher income and higher education were associated with telehealth use. Services were more often used for doctor’s appointment than physical, occupational, speech, or mental health therapies. Almost half (46%) of PwPD preferred to continue using telehealth always or sometimes after the coronavirus outbreak had ended. Having received support/instruction for telehealth and having a care partner, friend, or family member to help them with the telehealth visit increased the likelihood of continuous use of telehealth after the pandemic ended. Taken together, PD symptoms and management practices were markedly affected by COVID-19. Given the observed demographic limitations of telehealth, expanding its implementation to include additional physical, occupational, psychological, and speech therapies, increasing support for telehealth, as well as reaching underserved (low income) populations is urgently required.

## Introduction

The 2019 novel coronavirus (COVID-19) pandemic has affected the lives and daily routines of hundreds of millions of individuals across the world. People with Parkinson’s disease (PwPD), their care partners, and families are among those affected by SARS-CoV-2 infection and the mitigation strategies to control its spread^1^. Very little is known about the impact of public health policies implemented in the time of the pandemic on PwPD. PwPD are universally recommended to adhere to social distancing and all public health guidance. However, this is in contrast to the pre-pandemic PD management recommendations for increased socialization and physical activity^2–4^. Stress related psychiatric conditions, such as anxiety and depression, have been seen in 30–40% of PwPD prior to the pandemic^5^, and socialization with others has been successfully shown to minimize the occurrence of negative moods symptoms^4^. The importance of daily routines such as exercise for the attenuation of motor symptom progression in PwPD has also been well established^6,7^. In addition to public health restrictions that contrast with PD management recommendations, there have been profound changes in health care delivery. Treatment at health care institutions, routine physician care, as well as physical, occupational, and speech therapy visits have all been curtailed or modified by social distancing requirements^8,9^. Given the direct risks of COVID-19, the potential impact of social distancing recommendations on the access to health care services, exercise practices, and mental health in general, it is likely that PwPD have also been indirectly affected by the pandemic.

In order to provide guidance to clinicians, policy makers, and the PD community, there is a critical need to better understand how these factors have transformed the lives of PwPD and their health care delivery during the pandemic. Currently, a limited number of studies have shown the pandemic’s toll on the mental health and daily routines of PwPD^10–12^, specifically on worsening stress, anxiety (reported anxiety between 25% and 81% in PwPD), and depression^10^, (in a limited sample size). Our study aimed to test the social and emotional effect of the pandemic and social distancing on PwPD, and to explore the factors contributing to accessing alternative health care mechanisms, such as telehealth. We administered an anonymous knowledge, attitude, and practice (KAP) survey to 14,562 email addresses via the electronic mailing lists of the Parkinson’s Foundation and Columbia University Parkinson’s Disease Center of Excellence. Both PwPD and their care partners were invited to participate, resulting in 2,815 responses (response rate 19.3%). However, the current analysis was conducted including only 1,342 PwPD respondents from whom sufficient information was provided.

## Results

**Fig. 1:**
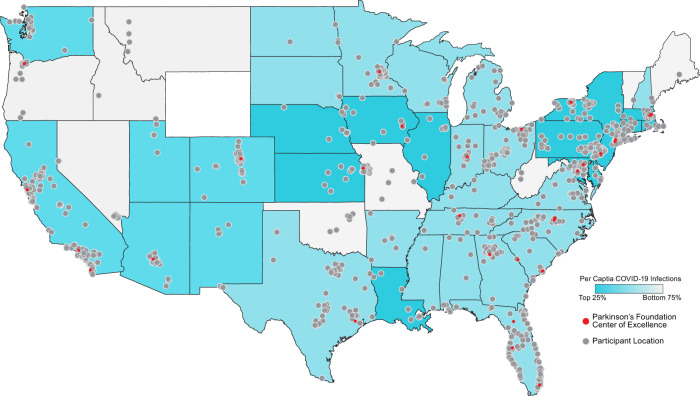
Participant representation by COVID-19 per capita infections as of May 27, 2020. US geographic location stratified by cumulative numbers of confirmed COVID-19 cases per capita (per 100,000) within that state as of May 27th, 2020; representing a total of 1,292 participants. Highest 25th percentile, >570 cases per 100,000; between 25th and 75th percentile, 200–750 cases per 100,000; lowest 75th percentile, <200 cases per 100,000. Darker states indicate higher per capita infections of COVID-19. Gray dots reflect survey participant locations. Red dots reflect the location of a Parkinson’s Foundation Center of Excellence, a medical center specializing in the care of people with Parkinson’s disease.

Almost all of the 1,342 PwPD respondents were located within the US and represented states from all levels of COVID-19 impact (96.3%, *n* = 1292). A third (31.1%, *n* = 418) of the PwPD respondents were from states within the highest quartile of cumulative confirmed COVID-19 cases per capita (>750 cases per 100,000) as of May 27, 2020 (Table 1 and Fig. 1). The mean age of PwPD respondents was 71 years old (range 32–93 years old). Half of respondents were female (679/50.6%). Over 90% (1216/1342) of PwPD self-identified as White, 2.4% reported more than one race (32/1342), and 2.4% self-identified as Hispanic Latino (32/1342). Most of the respondents received postsecondary education (93.7%, *n* = 1257), and were retired (75.3%, *n* = 1010). The PwPD respondents’ mean age at PD onset was 64 years old (range 21–91 years old) and had 7 years disease duration on average (range from <1 year to 52 years). About half of PwPD respondents (53.7%, *n* = 720) were taking L-dopa only and 36.1% (484/1342) were taking more than one PD medication. Only 17 (1.3%) PwPD reported having been diagnosed with COVID-19 by a health provider, of whom 5 had a COVID-19 diagnosis confirmed by testing. The most commonly reported COVID-19 symptoms included: fatigue (70.59%, *n* = 12), muscle pain (58.82%, *n* = 10), body aches (58.82%, *n* = 10), cough (62.94%, *n* = 9), headache (47.06%, *n* = 8), and shortness of breath (47.06%, *n* = 8) (Supplementary Table 1). The mean COVID-19 disease duration was 13.5 days.

**Table 1:** People with Parkinson’s disease respondent demographics (*n* = 1342).

	Case (percent)
Age (mean ± SD)^a^	70.9 ± 8.3
Disease duration (mean ± SD)^b^	7.0 ± 6.1
Age at onset (mean ± SD)^c^	63.9 ± 10.1
Gender	
Male	656 (48.9%)
Female	679 (50.6%)
Other	1 (0.1%)
Prefer to not answer	6 (0.4%)
Race	
American Indian or Alaska Native	3 (0.2%)
Asian	21 (1.6%)
Black/African–American	6 (0.4%)
Hispanic/Latino	32 (2.4%)
White	1216 (90.6%)
More than one race	32 (2.4%)
Other	8 (0.6%)
Prefer not to answer	24 (1.8%)
Postsecondary education	1257 (93.7%)
Income	
<$50,000	297 (22.1%)
$50–$100,000	394 (29.4%)
>$100,000	337 (25.1%)
Prefer not to answer	314 (23.4%)
Employment	
Not employed (unemployed or disability)	151 (11.3%)
Employed	181 (13.5%)
Retired	1010 (75.3%)
PD medication	
L-dopa^d^	720 (53.7%)
Dopamine agonists^d^	36 (2.7%)
MAO-B inhibitors^d^	22 (1.6%)
Other^d^	19 (1.4%)
Not taking any medication	60 (4.5%)
Taking more than one medication	484 (36.1%)
Participants located in the US	1292 (96.3%)
Geographic distribution by cumulative cases of COVID-19 infection per 100,000 at State level (in the US)	
<200	73 (5.5%)
200–570	801 (59.7%)
>570	418 (31.1%)
State/ Local restrictions (self-reported)	
Yes, a restriction is in effect	929 (69.2%)
No, a restriction was issued but has ended	247 (18.4%)
No, a restriction was never issued	82 (6.1%)
Not sure	84 (6.3%)
Self-reported COVID-19 diagnosis	
Self	17 (1.3%)
Know others who have been diagnosed	62 (4.6%)

*SD* standard deviation, *PD* Parkinson’s disease.

^a^Age was missing in four respondents.

^b^Disease duration was missing in nine respondents.

^c^Age at onset was missing in 13 respondents.

^d^Monotherapy.

Most of the PwPD respondents (87.9%, *n* = 1180) answered all COVID-19 knowledge questions correctly (questions including populations at risk, symptoms, and recommended response; Supplementary Table 2). During the questionnaire responding period, the majority of PwPD respondents reported that a travel restriction was in effect (69.2%, *n* = 929) or was issued, but had ended by the time they responded to the survey (18.4%, *n* = 247).

More than 85% of PwPD felt that their personal life has changed during the COVID-19 pandemic. The most commonly shared PwPD concerns included “Being infected myself”, “Being at an increased risk of infection because of PD”, and “Being infected and requiring intensive hospital care” (Supplementary Table 3). PwPD also took several actions and precautions during the coronavirus outbreak including: canceling or postponing personal or social activities and doctor’s appointments; avoiding public spaces, gatherings, crowds, or eating at restaurants; obtaining a 3-month supply of PD or other prescription medications; praying; and wearing a mask or other face covering (Supplementary Table 4). Among PwPD respondents, 804 (59.9%) canceled a doctor’s appointment during COVID-19. Fifty-five percent (443/804) of these PwPD were provided with telehealth or virtual medical appointment options, and 38.2% (307/804) participated in a telehealth visit in place of the canceled appointment.

Nearly half (45.9%, *n* = 616) of PwPD noticed some negative change in their Parkinson’s symptoms during the pandemic. However, most PwPD reported no negative change in their relationship with members of their household (64.6%, *n* = 867) or the frequency of their communication with others (74.4%, *n* = 999). The most common sources of social support were from individuals in their household (71.3%, *n* = 957), and friends or family via phone/video (48.7%, *n* = 653) or in-person visits (14.5%, *n* = 195).

### Telehealth

Out of all PwPD respondents, only 131 (9.7%) had used telehealth before the COVID-19 pandemic. During the COVID-19 pandemic (until June 11th, 2020), 63.5% of PwPD (852/1342) reported having used some type of telehealth service (Table 2). Among them, the most often used types of telehealth services were doctor’s or medical provider’s appointments (91.2%, *n* = 777), mental health therapy (19%, *n* = 162), and physical therapy (16.7%, *n* = 142). Among those who had used telehealth services during the pandemic, 46% (392/852) responded that they would prefer to continue using telehealth always or sometimes after the coronavirus outbreak had ended.

**Table 2:** Telehealth use characteristics in people with Parkinson’s disease.

	Case (percent)
Telehealth use	852 (63.5%)
Type of telehealth service used^a^	
Doctor or medical provider	777 (91.2%)
Physical therapist	142 (16.7%)
Occupational therapist	32 (3.8%)
Speech and language pathologist	67 (7.9%)
Mental health therapist	162 (19%)
Prior to the coronavirus outbreak, had you used telehealth or virtual medical appointments before?	
Yes	131 (9.7%)
No	1198 (89.3%)
Not sure	13 (1.0%)
How frequently would you prefer to continue telehealth or virtual medical appointments after the coronavirus outbreak has ended?^b^	
Always	25 (2.9%)
Sometimes	367 (43.1%)
Rarely	340 (39.9%)
Never	120 (14.1%)
Were you provided support or instructions on how to use telehealth or virtual services for this appointment?^c^	
I was provided support (by medical staff or someone familiar with technology)	198 (23.5%)
I was provided instructions	283 (33.6%)
I was provided both support and instructions	205 (24.3%)
I was not provided support or instruction	156 (18.5%)
Has a care partner, friend, or family member been helping you with your telehealth or virtual medical appointments?^c^	
Yes, holding the phone or device for me	20 (3.6%)
Yes, helping me with the technology (logging in, setting up audio or video, etc.)	145 (17.2%)
Yes, communicating with my doctor for or with me	87 (10.3%)
Yes, taking notes while my doctor is speaking	69 (8.2%)
Yes, other	24 (2.9%)
No, I am attending telehealth or virtual medical appointments on my own with no assistance	572 (67.9%)

^a^Percentage were calculated out of 852 total; cases included those who used multiple types of services.

^b^Only those who had used telehealth during the COVID-19 pandemic were asked to answer these questions; percentage were calculated out of 852 total.

^c^Ten respondents were excluded due to inconsistency between responses in the support and help questions.

Telehealth service was most commonly used to see a doctor or a medical provider. These respondents reported telehealth use for at least one appointment, and visits via video app or smart phone (30.6%, *n* = 228), personal computer video (38.8%, *n* = 289), and phone audio (28.5%, *n* = 212) were all commonly used methods (Supplementary Table 5). Most of the telehealth visits were for PD-related issues, including routine PD visits (61.2%, *n* = 456) and urgent PD visits (3.5%, *n* = 26). PwPD were equally (54.6%, *n* = 407) or more (12.6%, *n* = 94) satisfied with their telehealth visit with a doctor compared to in-person visits, and 363 (48.7%) of them would likely use telehealth sometimes or always after the pandemic had ended. Other services, including physical therapy, occupational therapy, speech and language pathology, and mental health therapy were less commonly used via telehealth.

Next, we aimed to identify predictors of usage of telehealth. In an adjusted logistic regression, telehealth use during the pandemic was associated with a household income >$100,000 per year (odds ratio (OR) 1.54, 95% confidence interval (CI) 1.06-1.76), PwPD who had received postsecondary education (OR 2.05, 95% CI 1.16–3.62), and those who had used telehealth prior to the COVID-19 pandemic (OR 2.27, 95% CI 1.34–3.85; Table 3). PwPD respondents who had felt depressed or hopeless were more likely to have used telehealth (OR 1.27, 95% CI 1.01–1.58), but the effect did not remain significant after adjusting for other variables. When compared to PwPD who were unemployed or on disability, employed PWPD were less likely to use telehealth services (OR 0.46, 95% CI 0.26–0.81). There were no utilization differences between retired and unemployed or on disability PwPD.

**Table 3:** Adjusted analysis using telehealth use during COVID-19 pandemic as outcome.

Predictors	Crude^a^		Multivariable^b^	
	OR (95% CI)	*P* value	OR (95% CI)	*P* value
Age	—	—	0.31 (0.03–3.33)	0.331
Disease duration	—	—	3.30 (0.30–36.02)	0.327
Age at onset	—	—	3.27 (0.30–35.58)	0.330
Taking L-dopa	1.15 (0.81–1.62)	0.472	0.89 (0.57–1.39)	0.599
Received postsecondary education	1.85 (1.19–2.88)	0.007	2.05 (1.16–3.62)	0.013
Gender, female	0.84 (0.68–1.06)	0.139	0.95 (0.72–1.26)	0.736
Race, White	1.08 (0.74–1.57)	0.771	1.08 (0.66–1.76)	0.757
Income				
<50k	Reference			
50–100k	1.05 (0.78–1.44)	0.721	1.11 (0.79–1.56)	0.558
>100k	1.40 (1.00–1.94)	0.047	1.54 (1.06–2.24)	0.025
Employment				
Unemployed	Reference			
Employed	0.54 (0.34–0.85)	0.007	0.46 (0.26–0.81)	0.007
Retired	0.75 (0.52–1.09)	0.130	0.71 (0.42–1.20)	0.205
Self-reported COVID-19 diagnosis	1.06 (0.39–2.87)	1.000^c^	1.00 (0.29–3.43)	0.994
State (cases per 100,000)				
<200	Reference			
200–570	1.10 (0.67–1.79)	0.704	1.02 (0.57–1.82)	0.951
>570	1.43 (0.86–2.38)	0.168	1.25 (0.68–2.28)	0.476
Nervous/anxious	1.14 (0.90–1.44)	0.280	0.82 (0.56–1.20)	0.307
Worry	1.20 (0.96–1.50)	0.124	1.10 (0.76–1.60)	0.606
Depressed/hopelessness	1.27 (1.01–1.58)	0.041	1.36 (0.93–1.97)	0.109
Little interest in doing things	1.21 (0.97–1.51)	0.099	1.14 (0.80–1.62)	0.483
Sleep interruption	1.13 (0.89–1.42)	0.338	1.07 (0.78–1.46)	0.690
Isolated	1.16 (0.93–1.45)	0.211	1.11 (0.80–1.55)	0.525
COVID-19 knowledge score	—	—	0.80 (0.54–1.20)	0.277
Had used telehealth before the pandemic	2.15 (1.40–3.31)	<0.001	2.27 (1.34–3.85)	0.002

*OR* odds ratio, *CI* confidence interval.

^a^Chi-square test.

^b^Logistic regression.

^c^Fisher’s exact.

Among PwPD, the preference for continued use of telehealth services after the COVID-19 pandemic (always or sometimes) was associated with the use of telehealth service before the pandemic (OR 2.30, 95% CI 1.47–3.59), having received support/instruction for telehealth (OR 1.51, 95% CI 1.03–2.21), and having a care partner, friend, or family member to help them with the telehealth visit (OR 1.40, 95% CI 1.03–1.91; Table 4). PwPD respondents that used telehealth for mental health services were less likely (OR 0.31, 95% CI 0.13–0.76) to consider using telehealth after the pandemic compared to those who used telehealth for doctor’s appointments.

**Table 4:** Adjusted analysis using interest in continuous use of telehealth after the COVID-19 pandemic ends (always or sometimes) as outcome.

Predictors	Crude^a^		Multivariable^b^	
	OR (95% CI)	*P* value	OR (95% CI)	*P* value
Age	—	—	5.26 (0.63–44.29)	0.127
Gender, female	1.06 (0.81–1.39)	0.729	1.11 (0.83–1.48)	0.486
Age at onset	—	—	0.19 (0.02–1.56)	0.121
Disease duration	—	—	0.19 (0.02–1.60)	0.126
Had used telehealth before the pandemic	2.32 (1.50–3.58)	<0.001	2.30 (1.47–3.59)	<0.001
Type of telehealth service used during the COVID-19 pandemic				
Doctor or medical provider	Reference			
Physical therapist	0.65 (0.28–1.50)	0.312	0.77 (0.32–1.87)	0.568
Occupational therapist^c^	—	—	1.41 (0.09–23.19)	0.808
Speech and language pathologist^c^	—	—	0.37 (0.07–1.84)	0.223
Mental health therapist	0.35 (0.15–0.83)	0.017	0.31 (0.13–0.76)	0.010
More than one service	1.16 (0.85–1.58)	0.360	1.11 (0.81–1.53)	0.529
Received support/instructions for telehealth	1.46 (1.02–2.08)	0.041	1.51 (1.03–2.21)	0.035
Had a care partner/friend/family helping with telehealth appointment	1.33 (0.99–1.77)	0.064	1.40 (1.03–1.91)	0.031

*OR* odds ratio, *CI* confidence interval.

^a^Chi-square test.

^b^Logistic regression.

^c^Limited number of observations.

### Physical and social activities

Almost half of PwPD (44.7%, *n* = 600) reported reduced hours of exercise, and a majority of respondents (72.9%, *n* = 978) reported a reduction in activities outside of their residence (e.g., exercise or wellness classes, support groups, recreational classes, educational events, etc.; Supplementary Table 6). Most (81.9%, *n* = 1099) reported that these activities were available in a virtual/online format, among whom 91.9% (999/1099) participated in those activities virtually/online (exercise/wellness class—58.4%, support groups—31.6%, recreational classes—12.5%, religious services—46.0%, educational events—35.5%, other—22.2%).

### Mood

More than half of respondents experienced nervousness or anxiety (66.5%, *n* = 893), feeling down or depressed (50.9%, *n* = 683), reduced interest or pleasure in doing things (53.7%, *n* = 720), or sleep disturbances (66.2%, *n* = 888) in the 6 weeks prior to the survey (since beginning of April 2020; Supplementary Table 7). Feelings of nervousness, anxiousness, or being on edge were associated with being female (OR 1.81, 95% CI 1.31–2.50), self-identification as White (OR 1.82, 95% CI 1.07–3.12), and having reduced exercise (OR 1.41, 95% CI 1.03–1.94). In contrast, a household income of >$100,000 per year (OR 0.63, 95% CI 0.41–0.98) and being retired compared to unemployed or having disability (OR 0.43, 95% CI 0.22–0.83) were inversely associated with feelings of nervousness or anxiousness. Uncontrollable worry, depression or hopelessness, and isolation were associated with being female, a household income <$50,000 per year, and reduced exercise (Supplementary Table 8). Sleep disturbances were associated with being female (OR 1.49, 95% CI 1.08–2.05) and Levodopa use (OR 2.23, 95% CI 1.39–3.58).

Respondents that reported experiencing mood disturbances (anxiety, worry, depression, reduced interest, sleep disturbances, or isolation) for more than half the days or nearly every day were further prompted through open text questions to explain why they felt that disruptions caused by the coronavirus had contributed to these symptoms (Supplemental Table 9). Anxiety was most often attributed to the fear of respondents getting infected themselves (21.6%, 54/250) and the unknowns about when COVID-19 would be resolved (13.2%, 33/250). Depression was most often attributed to the inability to see or have physical contact with family and friends (15.9%, 20/126), and having a history a depression unrelated to COVID-19 (11.9%, 15/126). Loss of interest was most often attributed to not leaving the house (14.4%, 23/160), having lost interest and apathy prior to COVID-19 (13.8%, 22/160), and hopelessness or negative feelings about the future (13.1%, 21/160). Sleep disturbances were most often attributed to prior sleep disturbances not related to COVID-19 (35.7%, 99/277) or worry related to COVID-19 (34.3%, 95/277). Six common themes emerged from analysis of all mood symptoms and were presented in Table 5.

**Table 5:** Themes from analysis of all mood symptoms.

Themes that emerged from analysis of all mood symptoms included the attribution of COVID-19 to the following negative outcomes:
• A lack of physical contact with others
• Complications and interruptions to routines and PD management practices, including medical appointments, exercise, and social support
• General fear and worry about future health, family, and economic outcomes
• A loss of purpose, focus, and belonging
• A hesitation to make plans
• A lack of trust in media and political leadership

## Discussion

The results of this study describe emotional and behavioral changes accompanying the public health policies adopted during the SARS-CoV-2 pandemic in a large PD community, with the most notable shift in practice within the PwPD population being the widespread use of telehealth. In addition, the results of this study highlight specific demographic factors associated with the use of telehealth, as well as with a higher prevalence of mood changes in PwPD during the pandemic. In our cohort, telehealth use significantly increased from 9.7% to 63.5% during the COVID-19 pandemic. Considering the study was closed June 11, 2020, <3 months from the implementation of social distancing regulations, this increase demonstrates a very fast change in the behaviors and practices of telehealth use among PwPD.

Our cohort was very educated about COVID-19. The majority (88%) of PwPD responded correctly to all knowledge questions, confirming a previous study reporting a similar finding in a smaller PwPD population^13^. Only 1.3% of PwPD reported a COVID-19 diagnosis and even fewer (0.4%) had their diagnosis confirmed by a test. The commonly reported COVID-19 symptoms by PwPD, including fatigue, muscle pain, body aches, and cough, were similar to findings among the general population^14,15^, although a larger sample size would be required to determine the prevalence of clinical characteristics or risk for COVID-19 among PwPD.

Not surprisingly, about half of respondents reported a reduction in exercise, and the majority reported a reduction in activities outside of their residence, though a vast majority indicated that such activities were available in an online format. As previously reported, depressed mood and anxiety symptoms were prevalent in PwPD during the pandemic^4–6^. Household income and employment status were found to be important factors in predicting negative mood, with nervousness and anxiousness negatively associated with being retired, while uncontrollable nervousness and anxiousness, worry, depression or hopelessness, and isolation were more likely to be present in PwPD with an annual household income <$50,000. Low socioeconomic status (income, education, and occupation) put individuals at higher risk of being depressed in pre-pandemic times^16^, and during the pandemic similar trends have been observed in the general population^17,18^. Our data strongly suggest disparities across socioeconomic status in PwPD during the COVID-19 pandemic, with those of lower income far more affected by financial and vocational uncertainties. Upon delving into the themes that emerged in the open text questions, those who reported more frequent disturbances in mood attributed such changes to various factors, including general fear and worry about future health, family, and economic outcomes. Similarly, a cross-sectional, case–control study from Italy found that fear of getting COVID-19 themselves and in family members were present in both PwPD and healthy controls, and was associated with increased severity of anxiety in PwPD^11^. Interestingly, factors such as geographic location and thus severity of COVID-19 outbreak in a given area were not associated with our measured mood symptoms, suggesting that communities deemed at lower risk of community spread are not immune from the overwhelming influence of the COVID-19 pandemic on everyday life.

Furthermore, about half of our respondents reported a negative change in PD symptoms, supporting previously published data regarding PD symptomatology during the pandemic^12,13^. However, it is not possible to determine from this survey whether PD symptoms indeed progressed, or if the reported negative changes could have resulted from worsening non-motor symptoms, such as depression and anxiety. One of the causes of stress among PwPD was the concern that COVID-19 may be more severe in PwPD. As it stands, there are inconsistent conclusions regarding mortality, hospitalization, and susceptibility in PwPD as compared to the general population^19–22^. Larger systematic studies are required to answer this question.

Within 3 months of social distancing regulation, nearly two thirds of our cohort utilized telemedicine for their care. Transitioning was not always easy; nevertheless, half of those using telemedicine wished to continue using it in the future. Formal studies of providers are lacking, but PwPD were clearly more likely to want to use telemedicine in the future if they received assistance for telehealth services, either from the health care providers or from their care partners (Table 4). These finding are consistent with previous reports on the importance of technical support for telemedicine^23–25^. Predictors for telemedicine usage in our cohort highlight that it was used by more affluent and educated PwPD, as both higher education and income were associated with telemedicine usage. Similar findings have been reported prior to the pandemic in 2014^26^. Based on our findings from the survey, we developed a series of recommendations for clinicians, for PwPD, and for health care systems and regulators shown in Table 6. Given the utility of telehealth services when in-person visits are reserved for emergencies, it is vital for stakeholders to develop mechanisms to make telemedicine more available to all appropriate prospective users. The COVID-19 pandemic has differentially impacted people from lower socioeconomic status^27^, and extending telemedicine services to economically disadvantaged populations may help reduce disparities. Furthermore, given the sparse representation of non-White ethnic groups in this study, further studies are needed to determine if utilization of these services are even more disparate in minority communities, in which lower socioeconomic status is more prevalent and has proven to be a barrier in health care service utilization and well-being^28–30^. To address these various disparities, technical support, education, and advertisement would all be required.

**Table 6:** Recommendations for better care during COVID-19.

Recommendations for clinicians
• Create a system to streamline pre-visit guidance and post-visit scheduling/referrals
• Provide PwPD with telehealth pre-visit checklist^a^
∘ Test system to be used a day prior to the visit
∘ Charge device to be used
∘ Start visit 15–20 min prior to appointment
∘ Obtain assistance from caregiver if needed
∘ Have medications/medication list available for review during the visit
∘ Sit in a quiet area
∘ Allow enough distance to the device for video clarity and comprehensive exam
∘ Identify unobstructed area to evaluate gait
• Targeted questions regarding: food, sleep, mood, and exercise (known to be more frequently affected per our survey; Supplementary Tables 4, 6, and 7)
• Identify links including resources for PwPD on exercise programs, support groups, and allied health providers offering remote care (>50% participated in these activities in a virtual format per our survey; Supplementary Table 6)
Recommendations for PwPD
• Follow your provider telehealth visit checklist as above^a^
• Ask for help from family members before the visit if required (significantly increases likelihood of continuous use of telehealth after the pandemic ends; Table 4)
• Look out for tele-resources, even outside your region/sate
Recommendations for health care systems and regulators
• Support the development of HIPPA compliant and user-friendly telehealth systems
• Provide pre-visit technical assistance (check in 1 day prior; significantly increases likelihood of continuous use of telehealth after the pandemic ends; Table 4)
• Continue the relaxation of regulation (e.g., seeing out of state providers and use of simple to use non-HIPPA compliant telehealth systems)
• Develop mechanisms to continue telemedicine post pandemic (>40% PwPD are interested; Table 2)

*PwPD* people with Parkinson’s disease.

^a^https://www.parkinson.org/blog/tips/Telemedicine.

More specific to PD, we observed that the use of telehealth for occupational, physical, and speech and language pathology are lagging behind physicians’ appointments. Considering our cohort’s report of reduced physical activity, expanding telehealth visits to these services is essential. In the height of the pandemic, many avoided hospital referrals even for emergencies. Further, physical and occupational therapy have been shown to help reduce falls, and speech and language pathology may reduce aspirations. Together, enhancing these services may reduce urgent care referrals and improve the well-being of PwPD.

Over 40% of the PwPD surveyed were interested in utilizing telemedicine after the pandemic. The ability to provide telehealth service during the pandemic was greatly facilitated by the lifting of cross-state practice restrictions, and reimbursement modifications. Stakeholders like foundations and advocacy groups should advocate on behalf of their community to regulators and legislators to help facilitate and maintain the availability of telemedicine even after the pandemic. Additional studies exploring the efficacy of telehealth visits (e.g., by reducing ER referrals) for new patient consultations and follow ups are required. Future studies should also investigate particular contributing factors to telehealth satisfaction rates in PwPD, with the ultimate target of optimizing remote services accordingly. Training for movement disorders specialists on how to conduct a visit and a movement disorders examination would further improve the quality of telemedicine visits.

A major strength of our study is the relatively large sample size (*n* = 1342), providing a great deal of power for the results. Our questionnaires were deployed in the height of the pandemic between May 2020 and June 2020, and did not require participants to retrospectively reflect on their actions and feelings. Indeed, this is the first study to describe telehealth use in PwPD during the COVID-19 pandemic. Our study, as is the case with many online questionnaires, was limited by a low response rate. We do not have data on how many of the email addresses were active. However, for comparison, a questionnaire on cannabis usage deployed to the Parkinson’ Foundation mailing list yielded 1,354 (17.8%) responses (compared to 2,815 [19.3%] here for both PwPD and care partners), signifying similar response rate. We do not have data on the demographic composition (e.g., age and gender) of people in the email lists, so we are unable to compare the demographics of responders to nonresponders. Furthermore, data was not captured from those who may have received an invitation to participate and passed away, from COVID-19 or other causes. Respondents to our survey comprised a convenience sample, and future studies with more representative samples will be needed to understand the effect of the pandemic on the greater PwPD community. The care partner perspective also remains unknown. Future studies will focus on the analysis of the perspective of the PwPD care partners.

In addition, the COVID-19 pandemic is a dynamic, and ever-changing situation, and as such data collected from respondents may have been affected by such changes during the time in which the survey was active. Given this was an electronic survey, it is likely that the technology and email requirements biased us toward those with better technological skill sets and access to the necessary technology. To reach out to additional populations that were not included in this study, we suggest that future studies distribute surveys in multiple languages (e.g., Spanish) to include a more diverse population. In addition, multiple recruitment methods should be considered in addition to email. For example, an ongoing study at Columbia University Irving Medical Center (CUIMC) aiming to understand the impact of COVID-19 on the local population is recruiting participants who identify as African–American/Hispanic and live in Upper Manhattan. Surveys were distributed to members of a Harlem church in addition to the CUIMC Neurology department listserv, with the option of completing the survey over the phone. The nature of an anonymous survey with reminders allowed for the inclusion of possible duplicate survey responses. For individuals that provided personal contact information, we were able to exclude duplicate responses, but it is possible that additional anonymous duplicates were undetected, and included in the data analysis. Lastly, it is important to note that some of the mood features reported in the survey may be associated with the phenotype of PD rather than the COVID-19 pandemic itself.

In summary, as the COVID-19 pandemic continues to unfold, it is essential to further explore the relationship between PD and COVID-19. Given the uncertainty of how long the hardship of COVID-19 and social distancing requirements may persist, it is crucial to identify mechanisms to help PwPD throughout this period. Gathering accurate data on COVID-19 risk among PwPD and improving telemedicine by ensuring a wider range of services (physical therapy, occupational therapy, speech and language pathology, and mental health therapy) are more accessible to all PwPD would be essential.

## Methods

### Standard protocol approvals, registrations, and patient consents

The CUIMC institutional review board (IRB) approved this study. Given the minimal risk to participants, the CUIMC IRB approved a waiver of written consent through a protocol-specific information sheet electronically presented to respondents prior to starting the survey. Study participants had the option of completing the questionnaire anonymously, or including their name if they wanted to be contacted for future studies. Participants who self-reported COVID-19 exposure were asked to share their contact information for future studies.

### Participants

We invited two separate cohorts to complete an online survey; the first cohort consisted of Parkinson’s Foundation constituents who had attended an online or in-person Parkinson’s Foundation educational event or had called the Parkinson’s Foundation helpline in calendar year 2019 or 2020 (*n* = 13,129), and the second cohort consisted of constituents enrolled in the CUIMC, Department of Neurology mailing list (*n* = 1433). Both people living with PD and care partners were eligible to complete the survey. Potential study participants received an initial email invitation and two additional weekly reminder emails. The initial invitation was sent May 13, 2020, and the survey remained open until June 11, 2020.

### Questionnaire

A review of publicly available surveys related to the coronavirus pandemic was conducted, and relevant questions were used or modified to fit this survey^31–37^. The survey consisted of two versions, one for people living with PD and the other for care partners. The care partner survey was modified to collect information about both the care partner and the person living with PD that they were caring for. The survey was then reviewed by five PwPD and one care partner to ensure relevance and readability. The complete questionnaire is available in Supplementary Methods.

The online questionnaire consisted of seven parts:

- Directory and screening questions to direct respondents to the correct version of the survey (*n* = 2).
- Diagnostic questions related to COVID-19 status and associated outcomes for themselves or individuals with which they were in close contact (*n* = 12 for PwPD; *n* = 13 for care partners).
- Knowledge questions regarding the COVID-19 outbreak and CDC recommended responses (*n* = 4).
- Attitudes questions related to fear surrounding COVID-19 and mental health (*n* = 7 for PwPD; *n* = 6 for care partners).
- Practice questions related to disruptions of daily activities and actions or precautions taken during the COVID-19 outbreak (*n* = 9 for PwPD; *n* = 10 for care partners).
- Questions related to the utilization of and satisfaction with virtual programs and telemedicine throughout the COVID-19 outbreak (*n* = 13).
- Basic demographics questions, including gender, year of birth and PD diagnosis, race/ethnicity, marital status, employment status, location of residence, etc. (*n* = 15 for PwPD; *n* = 14 for care partners).

**Fig. 2:**
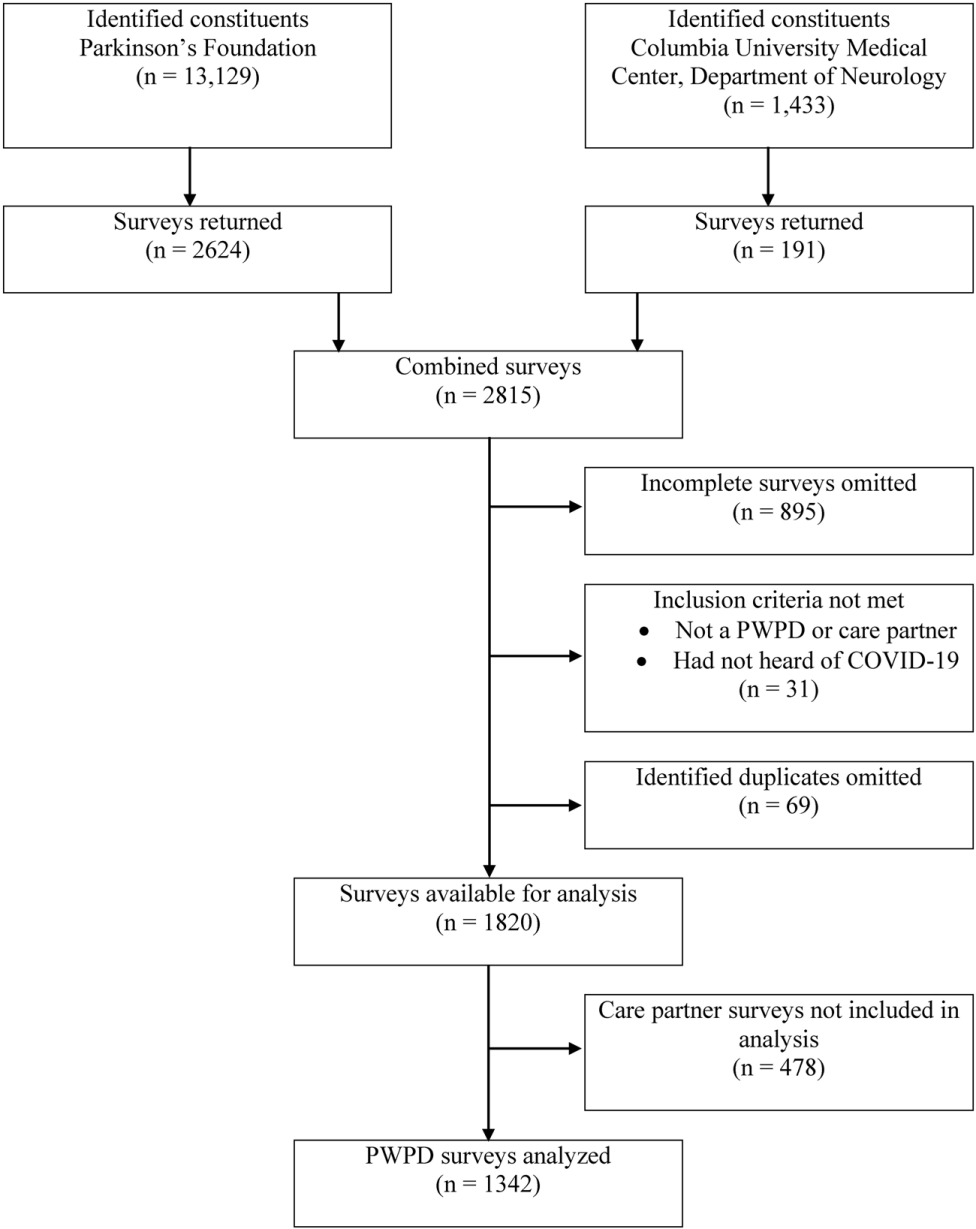
Flowchart of data cleaning approach. PwPD people with Parkinson’s disease.

There were two inclusion criteria for this study. Respondents needed to identify either as a PwPD or a care partner, and respondents had to have heard of COVID-19. If respondents identified as “other” or reported not having heard about COVID-19, they were excluded from the study. Of the 2,815 surveys returned from both cohorts combined, 895 were incomplete, and 31 did not meet inclusion criteria and were discarded from further analysis. There were 1,889 completed responses, however, after deduplication of the two cohorts, 1,820 completed responses were available for analysis (*n* = 1342 people living with PD and *n* = 478 care partners; Fig. 2).

### Statistical analyses

Descriptive statistics were used to summarize survey responses from PwPD only (frequency and percentage for categorical variables, mean and standard deviation (SD) for continuous variables). Hypothesizing that COVID-19 impact is associated with the level of exposure to COVID-19, we stratified respondents’ US geographic location into three group by cumulative numbers of confirmed cases per capita (per 100,000) within that state as of May 27th, 2020^38,39^. We compared demographics, telehealth characteristics, and KAP responses between telehealth use (Yes/No), likelihood to continue telehealth use after the COVID-19 outbreak ended (Yes/No), and experienced mood (Yes/No), using Chi-square tests (Fisher’s exact test when applicable) for categorical variables and Student’s *t* test for continuous variables. Logistic regression was performed in the above-mentioned groups adjusting for demographics, disease characteristics, telehealth responses, and KAP characteristics where applicable. More specifically, the logistic regression on telehealth use (Yes/No) were adjusted for age, gender, race, income, employment, PD disease duration, age at PD onset, taking L-dopa (Yes/No), self-reported COVID-19 diagnosis, geographic location (state by cases per 100,000), mood changes, computed COVID-19 knowledge score, and whether had used telehealth before the pandemic (Table 3). The logistic regression on continuous use of telehealth after the COVID-19 pandemic ends (always or sometimes vs. rarely or never) was adjusted for age, gender, PD disease duration, age at PD onset, whether had used telehealth before the pandemic, type of telehealth service used during the pandemic, whether received support/instructions for telehealth, and whether a care partner/friend/family had helped with the telehealth appointment (Table 4). Logistic regressions on mood (any vs. absence) were adjusted for age, gender, race, income, employment, PD disease duration, age at PD onset, taking L-dopa (Yes/No), geographic location (state by cases per 100,000), mood changes, computed COVID-19 knowledge score, exercise reduction (Yes/No), activity reduction (Yes/No), and whether participated in activities online (Supplementary Table 8). Open text collected in the survey were evaluated through thematic analysis. Statistical analysis was performed using SPSS 25.0 (SPSS Inc., Chicago, IL, USA), and data visuals were created using the R software package and programming language through RStudio desktop version 1.2.5042^40,41^.

### Reporting summary

Further information on research design is available in the Nature Research Reporting Summary linked to this article.

## Supplementary information

Supplementary material

Reporting summary

## Data Availability

Statistical analysis was performed using SPSS 25.0 (SPSS Inc., Chicago, IL, USA). SPSS syntax used for the analysis is available from the corresponding author upon reasonable request.

## Data availability

The datasets generated during and/or analyzed during the current study are available from the corresponding author on reasonable request.
