# Supplementary material for: The impact of COVID-19 and social distancing on people with Parkinson’s disease: a survey study
Source: NPJ Parkinsons Dis. 2021 Jan 21;7:10. doi: 10.1038/s41531-020-00153-8 (PMC7820020; doi:10.1038/s41531-020-00153-8)
Supplement: Supplementary file 1 — Supplementary material [file 41531_2020_153_MOESM1_ESM.pdf]

Supplementary material

Index

Supplementary Table 1. Demographics and disease characteristics of people with Parkinson's disease with self-reported COVID-19 diagnosis.

Supplementary Table 2. COVID-19 knowledge in respondents with Parkinson's disease.

Supplementary Table 3. Concerns related to COVID-19.

Supplementary Table 4. Changes in behavior due to COVID-19.

Supplementary Table 5. Service characteristics and satisfaction of telehealth services used by type.

Supplementary Table 6. Changes in exercise and activities, and participation in virtual activities.

Supplementary Table 7. Self-reported mood in people with Parkinson's disease.

Supplementary Table 8. Adjusted logistic regression on mood.

Supplementary Table 9. Most commonly identified open text mood topics.

Supplementary Methods - Parkinson's disease COVID-19 questionnaire for people with Parkinson's disease

Supplementary Table 1. Demographics and disease characteristics of people with Parkinson's disease with self-reported COVID-19 diagnosis.

		Case (Percent)
**Reported COVID-19 diagnosis**		17 (1.3%)
**Age (Mean ± SD)**		70.6 ± 10.8
**Disease Duration (Mean ± SD)**		6.5 ± 4.7
**Age at onset (Mean ± SD)**		64.5 ± 11.6
**Gender-Female**		9 (52.9%)
**Have you had a COVID-19 test to confirm this diagnosis?**		
	Yes, test positive for COVID-19	5 (29.4%)
	Yes, test negative for COVID-19	4 (23.5%)
	yes, waiting results	1 (5.9%)
	Not tested	7 (41.2%)
**Have you been in communication with a doctor or medical provider to monitor your COVID-19 symptoms?**		
	Yes	13 (76.5%)
	No	3 (17.7%)
	Not sure	1 (5.9%)
**Symptoms**		
	Fever	6 (35.3%)
	Chills	7 (41.2%)
	Cough	9 (52.9%)
	Shortness of breath	8 (47.1%)
	Sore throat	6 (35.3%)
	Diarrhea	3 (17.7%)
	Vomiting	3 (17.7%)
	Temporary loss of smell	5 (29.4%)
	Temporary loss of taste	4 (23.5%)
	Fatigue	12 (70.6%)
	Muscle pain	10 (58.8%)
	Body aches	10 (58.8%)
	Headache	8 (47.1%)
	Confusion/change in behavior	1 (5.9%)
	None	2 (11.8%)
	Not sure	1 (5.9%)
**Duration (Mean ± SD)**		13.5 ± 6.9
**Recovered**		
	Yes	10 (58.8%)
	No	3 (17.7%)
	Not sure	4 (23.5%)
**Had other people in close contact diagnosed with COVID-19**		4 (23.5%)

SD, standard deviation.

Supplementary Table 2. COVID-19 knowledge in respondents with Parkinson's disease.

		Correct (Percent)
**Who can get the COVID-19? - Anyone**		1308 (97.5%)
**Does everybody who has COVID-19 show symptoms? - No**		1286 (95.8%)
**As far as you know, if someone thinks they are having COVID-19 symptoms, what should they do? - Stay home and call a doctor or medical provider**		1257 (93.7%)
**Knowledge score (Number of correct answers)**		
	0	1 (0.1%)
	1	11 (0.8%)
	2	150 (11.2%)
	3	1180 (87.9%)

Supplementary Table 3. Concerns related to COVID-19.

Case (percent)<br>Type of concerns	To a great extent	Somewhat	Very little	Not at all	Don't know, N/A
Being infected myself	426 (31.7%)	617 (46.0%)	225 (16.8%)	67 (5.0%)	7 (0.5%)
Being at an increased risk of infection because of my Parkinson's	291 (21.7%)	474 (35.3%)	346 (25.8%)	212 (15.8%)	19 (1.4%)
Being infected and requiring intensive hospital care (mechanical ventilation or a breathing machine)	516 (38.5%)	442 (32.9%)	260 (19.4%)	113 (8.4%)	11 (0.8%)
Being infected and dying	418 (31.1%)	405 (30.2%)	333 (24.8%)	173 (12.9%)	13 (1.0%)
Requiring hospitalization for other non-COVID-19 related health complications	209 (15.6%)	434 (32.3%)	431 (32.1%)	255 (19.0%)	13 (1.0%)
Not being able to social distance because of required assistance due to Parkinson's	51 (3.8%)	205 (15.3%)	373 (27.8%)	674 (50.2%)	39 (2.9%)
Running out of Parkinson's medications	75 (5.6%)	198 (14.8%)	344 (25.6%)	707 (52.7%)	18 (1.3%)
Not having enough money for living expenses	77 (5.7%)	184 (13.7%)	325 (24.2%)	736 (54.8%)	20 (1.5%)
How long it will take for things to go back to normal	487 (36.3%)	610 (45.5%)	176 (13.1%)	46 (3.4%)	23 (1.7%)

Supplementary Table 4. Changes in behavior due to COVID-19.

Change in behavior / Case (percent)	Yes	No	Not Applicable
Canceled or postponed air travel for work or pleasure	576 (42.9%)	247 (18.4%)	519 (38.7%)
Canceled or postponed personal or social activities	1184 (88.2%)	90 (6.7%)	68 (5.1%)
Visited a doctor	517 (38.5%)	738 (55.0%)	87 (6.5%)
Canceled a doctor's appointment	804 (59.9%)	473 (35.2%)	65 (4.8%)
Stockpiled food or water	544 (40.5%)	761 (56.7%)	37 (2.8%)
Avoided contact with people who could be high-risk	1122 (83.6%)	153 (11.4%)	67 (5.0%)
Avoided public spaces, gatherings, or crowds	1264 (94.2%)	58 (4.3%)	20 (1.5%)
Avoided eating at restaurants	1242 (92.5%)	55 (4.1%)	45 (3.4%)
Prayed	860 (64.1%)	368 (27.4%)	114 (8.5%)
Obtained a three-month supply of Parkinson's prescription medications	835 (62.4%)	451 (33.7%)	53 (4%)
Obtained a three-month supply of other essential prescription medications	725 (54.0%)	538 (40.1%)	79 (5.9%)
Worked or studied at home	521 (38.8%)	300 (22.4%)	521 (38.8%)
Worn a mask or other face covering	1260 (93.9%)	76 (5.7%)	6 (0.4%)
Has anything prevented you from following the CDC and government social distancing and coronavirus health recommendations?	43 (3.2%)	1299 (96.8%)	

Supplementary Table 5. Service characteristics and satisfaction of telehealth services used by type.

	Case (Percent)				
	**Doctor or medical provider**	**Physical therapist**	**Occupational therapist**	**Speech and language pathologist**	**Mental health therapist**
**Number of observations [a]**	745	124	25	57	150
**Number of appointments**					
1 appointment	439 (58.9%)	46 (37.1%)	6 (24.0%)	19 (33.3%)	48 (32.0%)
2 appointments	191 (25.6%)	17 (13.7%)	6 (24.0%)	6 (10.5%)	42 (28.0%)
3-5 appointments	103 (13.8%)	23 (18.5%)	5 (20.0%)	14 (24.6%)	36 (24.0%)
>=6 appointments	12 (1.6%)	38 (30.6%)	8 (32.0%)	18 (31.6%)	24 (16.0%)
**How was this telehealth or virtual appointment with a doctor of medical provider conducted?**					
Video- application or smart phone	228 (30.6%)	23 (18.5%)	5 (20.0%)	[b]	33 (22.0%)
Video- personal computer	289 (38.8%)	68 (54.8%)	9 (36.0%)	[b]	51 (34.0%)
Phone audio	212 (28.5%)	18 (14.5%)	3 (12.0%)	[b]	60 (40.0%)
Other	16 (2.2%)	15 (12.1%)	8 (32.0%)	[b]	6 (4.0%)
**Satisfaction (comparing with in-person visits)**					
More satisfied	94 (12.6%)	23 (18.5%)	9 (36.0%)	13 (22.8%)	19 (12.7%)
Equally satisfied	407 (54.6%)	52 (41.9%)	7 (28.0%)	29 (50.9%)	80 (53.3%)
Less satisfied	226 (30.3%)	42 (33.9%)	8 (32.0%)	11 (19.3%)	42 (28.0%)
Not sure	18 (2.4%)	7 (5.6%)	1 (4.0%)	4 (7.0%)	9 (6.0%)
**How frequently would you prefer to continue telehealth or virtual medical appointments after the coronavirus outbreak has ended?**					
Always	20 (2.7%)	7 (5.6%)	4 (16.0%)	4 (7.0%)	6 (4.0%)
Sometimes	343 (46.0%)	53 (42.7%)	5 (20.0%)	24 (42.1%)	63 (42.0%)
Rarely	292 (39.2%)	48 (38.7%)	10 (40.0%)	23 (40.4%)	63 (42.0%)
Never	90 (12.1%)	16 (12.9%)	6 (24.0%)	6 (10.5%)	18 (12.0%)
**Were any of your telehealth or virtual appointments related to any of the following medical issues?**					
Routine PD visit	456 (61.2%)				
Urgent PD visit	26 (3.5%)				
COVID-19 symptom visit	23 (3.1%)				
Other urgent visit	101 (13.6%)				
None of the above	197 (26.4%)				

a. Number displayed included those who used more than one type of telehealth service.
b. Data missing because of an error in questionnaire logic.
PD, Parkinson's disease.

Supplementary Table 6. Changes in exercise and activities, and participation in virtual activities.

		Case (Percent)
**Change in exercise**		
	Reduced	600 (44.7%)
	Unchanged	626 (46.6%)
	Increased	116 (8.6%)
**Change in activities outside of residence**		
	Reduced	978 (72.9%)
	Unchanged	313 (23.3%)
	Increased	51 (3.8%)
**Have activities available in a virtual/online format**		1099 (81.9%)
**Participated in those activities in a virtual/online format?** [a]		999 (90.9%)
**What activities have you participated in a virtual/online format?** [b]		
	Exercise or wellness class	583 (58.4%)
	Support groups	316 (31.6%)
	Recreational classes (art, dance, drama, etc.)	125 (12.5%)
	Religious services	460 (46.0%)
	Educational events	355 (35.5%)
	Other	222 (22.2%)

a. Percentage out of 1099 total.
b. Percentage out of 999 total.

Supplementary Table 7. Self-reported mood in people with Parkinson's disease.

	Any			Not at all
	**Nearly everyday**	**More than half the days**	**Several days**	
**Feeling nervous, anxious, or on edge**	153 (11.4%)	188 (14.0%)	552 (41.1%)	449 (33.5%)
**Not being able to stop or control worrying**	78 (5.8%)	132 (9.9%)	395 (29.4%)	737 (54.9%)
**Feeling down, depressed, or hopeless**	80 (6.0%)	108 (8.1%)	494 (36.9%)	659 (49.1%)
**Little interest or pleasure in doing things**	80 (6.0%)	141 (10.5%)	499 (37.2%)	622 (46.3%)
**Had sleep interruptions or disturbances**	149 (11.1%)	216 (16.1%)	523 (39.0%)	454 (33.8%)
**Feeling isolated, left out or alone**	83 (6.2%)	112 (8.4%)	419 (31.2%)	728 (54.2%)

Supplementary Table 8. Adjusted logistic regression using mood as outcome (any vs. absence).

Predictors		Nervous/anxious		Worry		Depressed		No interest		Sleep disturbance		Isolated	
		OR (95%CI)	P	OR (95%CI)	P	OR (95%CI)	P	OR (95%CI)	P	OR (95%CI)	P	OR (95%CI)	P
**Age**		2.31 (0.25-21.52)	0.462	0.65 (0.07-5.80)	0.696	1.42 (0.16-12.70)	0.754	0.41 (0.04-4.07)	0.449	1.58 (0.17-15.18)	0.690	0.63 (0.07-5.79)	0.686
**Disease duration**		0.43 (0.05-4.00)	0.456	1.53 (0.17-13.86)	0.703	0.7 (0.08-6.26)	0.747	2.4 (0.24-23.77)	0.454	0.62 (0.06-5.98)	0.680	1.55 (0.17-14.21)	0.699
**Age at onset**		0.43 (0.05-4.03)	0.462	1.56 (0.17-14.04)	0.692	0.71 (0.08-6.32)	0.755	2.41 (0.24-23.76)	0.452	0.62 (0.06-5.90)	0.673	1.55 (0.17-14.17)	0.698
**Taking L-dopa**		1.2 (0.73-1.99)	0.478	1.33 (0.82-2.14)	0.250	1.48 (0.92-2.38)	0.107	1.63 (1.02-2.62)	0.042	2.23 (1.39-3.58)	0.001	1.52 (0.93-2.47)	0.092
**Received post-secondary education**		0.94 (0.44-2.04)	0.882	0.78 (0.40-1.51)	0.460	0.61 (0.30-1.22)	0.164	0.71 (0.35-1.43)	0.341	0.98 (0.46-2.05)	0.948	1.38 (0.71-2.69)	0.348
**Gender- Female**		1.81 (1.31-2.50)	<0.001	1.56 (1.16-2.10)	0.003	1.48 (1.10-1.98)	0.010	1.3 (0.97-1.75)	0.080	1.49 (1.08-2.05)	0.015	1.59 (1.19-2.14)	0.002
**Race-White**		1.82 (1.07-3.12)	0.029	0.85 (0.51-1.43)	0.537	0.8 (0.47-1.36)	0.408	1.31 (0.78-2.21)	0.308	0.98 (0.56-1.73)	0.949	0.92 (0.54-1.55)	0.744
**Income**	<50k	Reference											
	50-100k	0.83 (0.54-1.26)	0.371	0.68 (0.47-0.99)	0.043	0.8 (0.55-1.16)	0.234	0.83 (0.57-1.21)	0.338	0.82 (0.55-1.24)	0.355	0.78 (0.54-1.14)	0.197
	>100k	0.63 (0.41-0.98)	0.039	0.58 (0.40-0.86)	0.007	0.53 (0.36-0.79)	0.002	0.59 (0.40-0.88)	0.009	0.7 (0.46-1.08)	0.107	0.53 (0.36-0.79)	0.002
**Employment**	Unemployed	Reference											
	Employed	0.61 (0.30-1.25)	0.177	0.62 (0.34-1.13)	0.116	0.86 (0.47-1.55)	0.606	0.53 (0.29-0.96)	0.037	0.79 (0.40-1.56)	0.492	0.59 (0.32-1.08)	0.086
	Retired	0.43 (0.22-0.83)	0.013	0.59 (0.34-1.01)	0.052	0.99 (0.57-1.70)	0.961	0.69 (0.40-1.21)	0.196	0.81 (0.43-1.51)	0.503	0.74 (0.43-1.28)	0.278
**State (cases per 100,000)**	<200	Reference											
	200-750	0.71 (0.34-1.48)	0.361	0.67 (0.36-1.25)	0.208	1.03 (0.55-1.93)	0.939	0.73 (0.38-1.40)	0.347	1.09 (0.56-2.11)	0.805	0.97 (0.51-1.81)	0.915
	>750	0.68 (0.32-1.44)	0.314	0.78 (0.41-1.49)	0.455	0.85 (0.44-1.64)	0.633	0.69 (0.35-1.35)	0.280	1.07 (0.54-2.13)	0.841	1.02 (0.53-1.97)	0.946
**Reduced exercise**		1.41 (1.03-1.94)	0.034	1.38 (1.03-1.85)	0.030	1.36 (1.02-1.83)	0.037	1.49 (1.11-2.00)	0.008	1.16 (0.85-1.58)	0.366	1.5 (1.12-2.01)	0.006
**Reduced activity**		1.21 (0.85-1.72)	0.302	1.15 (0.82-1.61)	0.412	1.4 (1.00-1.95)	0.052	1.06 (0.76-1.49)	0.727	1.35 (0.95-1.92)	0.093	1.33 (0.95-1.87)	0.102
**Used telehealth**		0.97 (0.70-1.35)	0.862	1.18 (0.87-1.60)	0.279	1.32 (0.97-1.79)	0.076	1.19 (0.88-1.62)	0.263	1.1 (0.79-1.52)	0.583	1.18 (0.87-1.61)	0.289
**Participated activities online**		0.99 (0.57-1.7)	0.962	0.86 (0.52-1.43)	0.556	1.18 (0.71-1.98)	0.524	0.76 (0.45-1.28)	0.309	1.54 (0.91-2.60)	0.104	0.99 (0.59-1.66)	0.978
**COVID-19 knowledge score**		1.34 (0.87-2.06)	0.185	1.01 (0.66-1.53)	0.976	1.5 (0.99-2.30)	0.059	1.36 (0.90-2.07)	0.146	0.73 (0.46-1.15)	0.175	1.09 (0.72-1.67)	0.677

Supplementary Table 9. Most commonly identified open text mood topics.

	Case (Percent)
**Feeling nervous, anxious, or on edge** (n = 250)	
Fear of getting infected	54 (21.6%)
The unknowns about when COVID-19 would be resolved	33 (13.2%)
Fear for family	29 (11.6%)
Inability to see or have physical contact family and friends	29 (11.6%)
**Not being able to stop or control worrying** (n = 140)	
The unknowns about when COVID-19 would be resolved	22 (15.7%)
Constant media coverage of COVID-19	18 (12.9%)
Lack of control/helplessness	18 (12.9%)
**Feeling down, depressed, or hopeless** (n = 126)	
Inability to see or have physical contact family and friends	20 (15.9%)
Having a history of depression unrelated to COVID-19	15 (11.9%)
Disruption or change in routines	9 (7.1%)
Inability to leave the house for social distancing reasons	9 (7.1%)
Isolation and loneliness	9 (7.1%)
**Little interest or pleasure in doing things** (n = 160)	
Inability to leave the house for social distancing reasons	23 (14.4%)
Having a history of lost interest or apathy unrelated to COVID-19	22 (13.8%)
General negative outlook or hopelessness	21 (13.1%)
**Had sleep interruptions or disturbances** (n = 277)	
Having a history of sleep issues unrelated to COVID-19	99 (35.7%)
Worry	95 (34.3%)
The unknowns about when COVID-19 would be resolved	14 (5.1%)
**Feeling isolated, left out or alone** (n = 138)	
Inability to see or have physical contact family and friends	49 (35.5%)
Isolation and loneliness	37 (26.8%)
Inability to participate in in-person social activities	28 (20.3%)

Parkinson's disease COVID-19 questionnaire

Variable / Field Name	Question	Choices, Calculations, OR Slider Labels	Branching Logic (Show field only if...)	Required Field?
diagnosis_1	Has a doctor or another healthcare professional diagnosed you or someone in close proximity to you as having or probably having COVID-19? Please select all that apply.	1, I was diagnosed as having or probably having COVID-19 | 2, My care partner was diagnosed as having or probably having COVID-19 | 3, Someone in my household or residence was diagnosed as having or probably having COVID-19 | 4, Someone who regularly supports me in my household such as a home health assistant or nursing assistant was diagnosed as having or probably having COVID-19 | 5, An immediate family member not in my household but with whom I am in close contact was diagnosed as having or probably having COVID-19 (can include spouse, parent, children, grandchildren, siblings and in-laws) | 6, A friend whom I am in close contact with was diagnosed as having or probably having COVID-19 | 7, No one was diagnosed as having or probably having COVID-19	[directory_1] = '1' and ([directory_2] = '1' or [directory_2] = '3')	y
diagnosis_1_1_pwp	Have you had a COVID-19 test to confirm this diagnosis?	1, Yes, tested and received a positive test result | 2, Yes, tested but received a negative test result | 3, Tested, but awaiting test results | 4, No, not tested | 5, Not sure	[diagnosis_1(1)] = '1'	y

diagnosis_1_1_cp	Has your care partner had a COVID-19 test to confirm this diagnosis?	1, Yes, tested and received a positive test result | 2, Yes, tested but received a negative test result | 3, Tested, but awaiting test results | 4, No, not tested | 5, Not sure	[diagnosis_1(2)] = '1'	y
diagnosis_1_1_household	Has this member of your household had a COVID-19 test to confirm this diagnosis?	1, Yes, tested and received a positive test result | 2, Yes, tested but received a negative test result | 3, Tested, but awaiting test results | 4, No, not tested | 5, Not sure	[diagnosis_1(3)] = '1'	y

diagnosis_1_1_assist	Has your home health assistant had a COVID-19 test to confirm this diagnosis?	1, Yes, tested and received a positive test result | 2, Yes, tested but received a negative test result | 3, Tested, but awaiting test results | 4, No, not tested | 5, Not sure	[diagnosis_1(4)] = '1'	y
diagnosis_1_1_family	Has this family member had a COVID-19 test to confirm this diagnosis?	1, Yes, tested and received a positive test result | 2, Yes, tested but received a negative test result | 3, Tested, but awaiting test results | 4, No, not tested | 5, Not sure	[diagnosis_1(5)] = '1'	y

diagnosis_1_1_friend	Has this friend had a COVID-19 test to confirm this diagnosis?	1, Yes, tested and received a positive test result | 2, Yes, tested but received a negative test result | 3, Tested, but awaiting test results | 4, No, not tested | 5, Not sure	[diagnosis_1(6)] = '1'	y
diagnosis_1_2_pwp	Have you been in communication with a doctor or medical provider to monitor your COVID-19 symptoms?	1, Yes | 2, No | 3, Not sure	[diagnosis_1(1)] = '1'	y
diagnosis_1_2_cp	Has your care partner been in communication with a doctor or medical provider to monitor their COVID-19 symptoms?	1, Yes | 2, No | 3, Not sure	[diagnosis_1(2)] = '1'	y
diagnosis_1_2_household	Has this member of your household been in communication with a doctor or medical provider to monitor their COVID-19 symptoms?	1, Yes | 2, No | 3, Not sure	[diagnosis_1(3)] = '1'	y
diagnosis_1_3_pwp	How have you been managing COVID-19? <span style="font-weight: normal; Please select all that apply.	1, Isolating at home | 2, Isolating in an assisted living facility | 3, Staying with family | 4, Through non-critical hospital care | 5, Through critical hospital care (in an intensive care unit (ICU) or on a ventilator) | 6, Passed away from COVID-19 or complications due to COVID-19 | 7, Other | 8, Not sure	[diagnosis_1(1)] = '1'	y
diagnosis_1_3_pwp_1	In what other way have you managed COVID-19?		[diagnosis_1_3_pwp(7)] = '1'	y
diagnosis_1_3_cp	How has your care partner been managing COVID-19? Please select all that apply.	1, Isolating at home | 2, Isolating in an assisted living facility | 3, Staying with family | 4, Through non-critical hospital care | 5, Through critical hospital care (in an intensive care unit	[diagnosis_1(2)] = '1'	y

		(ICU) or on a ventilator) | 6, Passed away from COVID-19 or complications due to COVID-19 | 7, Other | 8, Not sure		
diagnosis_1_3_cp_1	In what other way have they managed COVID-19?		[diagnosis_1_3_cp(7)] = '1'	y
diagnosis_1_3_household	How has this member of your household been managing COVID-19? Please select all that apply.	1, Isolating at home | 2, Isolating in an assisted living facility | 3, Staying with family | 4, Through non-critical hospital care | 5, Through critical hospital care (in an intensive care unit (ICU) or on a ventilator) | 6, Passed away from COVID-19 or complications due to COVID-19 | 7, Other | 8, Not sure	[diagnosis_1(3)] = '1'	y
diagnosis_1_3_household_1	In what other way have they managed COVID-19?		[diagnosis_1_3_household(7)] = '1'	y
diagnosis_1_4_pwp	Where did you first seek medical care for COVID-19? This can include reaching out in person or over the phone.	1, Hospital or emergency room | 2, Urgent care | 3, My primary care doctor or another doctor | 4, A local health department | 5, Other | 6, I did not seek medical care	[diagnosis_1(1)] = '1'	y
diagnosis_1_4_pwp_1	At what other place did you first seek medical care for COVID-19?		[diagnosis_1_4_pwp] = '5'	y
diagnosis_1_4_cp	Where did your care partner first seek medical care for COVID-19? This can include reaching out in person or over the phone.	1, Hospital or emergency room | 2, Urgent care | 3, Their primary care doctor or another doctor | 4, A local health department | 5, Other | 6, I did not seek medical care	[diagnosis_1(2)] = '1'	y
diagnosis_1_4_cp_1	At what other place did your care partner first seek medical care for COVID-19?		[diagnosis_1_4_cp] = '5'	y
diagnosis_1_4_household	Where did this member of your household first seek medical care for COVID-19? This can include reaching out in person or over the phone.	1, Hospital or emergency room | 2, Urgent care | 3, Their primary care doctor or another doctor | 4, A local health department | 5, Other | 6, I did not seek medical care	[diagnosis_1(3)] = '1'	y
diagnosis_1_4_cp_3	At what other place did this member of your household first seek medical care for COVID-19?		[diagnosis_1_4_household] = '5'	y
diagnosis_1_5_pwp	What COVID-19 symptoms did you experience? Please select all that apply.	1, Fever | 2, Chills | 3, Cough | 4, Shortness of breath | 5, Sore throat | 6, Diarrhea | 7, Vomiting | 8,	[diagnosis_1(1)] = '1'	y

		Temporary loss of smell | 9, Temporary loss of taste | 10, Fatigue | 11, Muscle pain | 12, Body aches | 13, Headache | 14, Confusion or change in behavior | 15, I had none of these symptoms | 16, Not sure		
diagnosis_1_5_cp	What COVID-19 symptoms did your care partner experience? Please select all that apply.	1, Fever | 2, Chills | 3, Cough | 4, Shortness of breath | 5, Sore throat | 6, Diarrhea | 7, Vomiting | 8, Temporary loss of smell | 9, Temporary loss of taste | 10, Fatigue | 11, Muscle pain | 12, Body aches | 13, Headache | 14, Confusion or change in behavior | 15, They had none of these symptoms | 16, Not sure	[diagnosis_1(2)] = '1'	y
diagnosis_1_5_household	What COVID-19 symptoms did this member of your household experience? Please select all that apply.	1, Fever | 2, Chills | 3, Cough | 4, Shortness of breath | 5, Sore throat | 6, Diarrhea | 7, Vomiting | 8, Temporary loss of smell | 9, Temporary loss of taste | 10, Fatigue | 11, Muscle pain | 12, Body aches | 13, Headache | 14, Confusion or change in behavior | 15, They had none of these symptoms | 16, Not sure	[diagnosis_1(3)] = '1'	y
diagnosis_1_6_pwp	For how many days have you experienced COVID-19 symptoms?	1, 1 | 2, 2 | 3, 3 | 4, 4 | 5, 5 | 6, 6 | 7, 7 | 8, 8 | 9, 9 | 10, 10 | 11, 11 | 12, 12 | 13, 13 | 14, 14 | 15, 15 | 16, 16 | 17, 17 | 18, 18 | 19, 19 | 20, 20 | 21, 21 | 22, 22 | 23, 23 | 24, 24 | 25, 25 | 26, 26 | 27, 27 | 28, 28 | 29, 29 | 30, 30 | 31, 30+ days | 32, Not sure	[diagnosis_1(1)] = '1'	y
diagnosis_1_6_cp	For how many days has your care partner experienced COVID-19 symptoms?	1, 1 | 2, 2 | 3, 3 | 4, 4 | 5, 5 | 6, 6 | 7, 7 | 8, 8 | 9, 9 | 10, 10 | 11, 11 | 12, 12 | 13, 13 | 14, 14 | 15, 15 | 16, 16 | 17, 17 | 18, 18 | 19, 19 | 20, 20 | 21, 21 | 22, 22 | 23, 23 | 24, 24 | 25, 25 | 26, 26 | 27, 27 | 28, 28 | 29, 29 | 30, 30 | 31, 30+ days | 32, Not sure	[diagnosis_1(2)] = '1'	y

diagnosis_1_6_household	For how many days has this member of your household experienced COVID-19 symptoms?	1, 1 | 2, 2 | 3, 3 | 4, 4 | 5, 5 | 6, 6 | 7, 7 | 8, 8 | 9, 9 | 10, 10 | 11, 11 | 12, 12 | 13, 13 | 14, 14 | 15, 15 | 16, 16 | 17, 17 | 18, 18 | 19, 19 | 20, 20 | 21, 21 | 22, 22 | 23, 23 | 24, 24 | 25, 25 | 26, 26 | 27, 27 | 28, 28 | 29, 29 | 30, 30 | 31, 30+ days | 32, Not sure	[diagnosis_1(3)] = '1'	y
diagnosis_1_7_pwp	Have you recovered from COVID-19?	1, Yes | 2, No | 3, Not sure	[diagnosis_1(1)] = '1'	y
diagnosis_1_7_cp	Has your care partner recovered from COVID-19?	1, Yes | 2, No | 3, Not sure	[diagnosis_1(2)] = '1'	y
diagnosis_1_7_household	Has this member of your household recovered from COVID-19?	1, Yes | 2, No | 3, Not sure	[diagnosis_1(3)] = '1'	y
diagnosis_1_8_pwp	To learn more about how the coronavirus effects the health of people with Parkinson's disease, can a member of the research team from Parkinson's Foundation or Columbia University call you with more detailed questions about your COVID-19 symptoms and health outcomes?	1, Yes | 2, No	[diagnosis_1(1)] = '1'	
diagnosis_1_9_pwp_1	Please provide your name, email and phone number so that we may follow-up with you.<br><br>Name:		[diagnosis_1_8_pwp] = '1'	
diagnosis_1_9_pwp_2	Email:		[diagnosis_1_8_pwp] = '1'	

diagnosis_1_9_pwp_3	Phone number:		[diagnosis_1_8_pwp] = '1'	
diagnosis_2	Have you tried to get tested for COVID-19?	1, Yes, and I was able to get tested | 2, Yes, but testing was not locally available | 3, Yes, but the doctor or provider would not allow me to get tested or did not feel that my symptoms were severe enough to get tested | 4, Yes, but I could not afford the cost of testing | 5, No, I did not try to get tested for COVID-19 | 6, Other	[directory_1] = '1' and ([directory_2] = '1' or [directory_2] = '3')	y
diagnosis_2_1	For what other reason were you unable to get tested for COVID-19?		[diagnosis_2] = '6'	y
diagnosis_3	To your knowledge, has a doctor ever told you that you had any of the following medical conditions?	1, Any heart disease or history of heart attack or stroke | 2, Any chronic lung disease (e.g., asthma, chronic obstructive pulmonary disease, COPD/emphysema/chronic bronchitis) | 3, Active/current cancer | 4, Hypertension (high blood pressure) | 5, Diabetes (high blood sugar) | 6, Severe obesity | 7, Any autoimmune disease (e.g., lupus, multiple sclerosis, rheumatoid arthritis, psoriasis, Crohn's disease, inflammatory bowel disease) | 8, Any depressive disorder (e.g., major depression) | 9, Any anxiety disorder (e.g., panic disorder, generalized anxiety disorder, post-traumatic stress disorder) | 10, None of the above medical conditions	[directory_1] = '1' and ([directory_2] = '1' or [directory_2] = '3')	y
knowledge_4	Who can get the COVID-19?	1, Older adults | 2, Young children | 3, People with underlying health conditions (heart disease, lung disease, etc.) | 4, Men | 5, Health care workers | 6, Anyone can get COVID-19	[directory_1] = '1' and ([directory_2] = '1' or [directory_2] = '3')	y
knowledge_5	Does everybody who has COVID-19 show symptoms?	1, Yes | 2, No | 3, Not sure	[directory_1] = '1' and ([directory_2] = '1' or [directory_2] = '3')	y

knowledge_6	As far as you know, if someone thinks they are having COVID-19 symptoms, what should they do?	1, Stay home and call a doctor or medical provider | 2, Seek care immediately at an ER or urgent care facility	[directory_1] = '1' and ([directory_2] = '1' or [directory_2] = '3')	y
knowledge_7	Are there travel restrictions in effect in your area? Restrictions can include stay-at-home, shelter-in-place, healthy-at-home, essential travel or curfew orders.	1, Yes, a restriction is in effect | 2, No, a restriction was issued but has ended | 3, No, a restriction was never issued | 4, Not sure	[directory_1] = '1' and ([directory_2] = '1' or [directory_2] = '3')	y
attitudes_8	As a result of the coronavirus, would you say your personal life has...	1, Changed in a major way | 2, Changed, but only a little bit | 3, Stayed about the same	[directory_1] = '1' and ([directory_2] = '1' or [directory_2] = '3')	y
attitudes_9_self	Being infected myself	1, To a Great Extent | 2, Somewhat | 3, Very Little | 4, Not at All | 5, I don't know/I prefer not to answer/Not applicable	[directory_1] = '1' and ([directory_2] = '1' or [directory_2] = '3')	y
attitudes_9_increased_risk	Being at an increased risk of infection because of my Parkinson's	1, To a Great Extent | 2, Somewhat | 3, Very Little | 4, Not at All | 5, I don't know/I prefer not to answer/Not applicable	[directory_1] = '1' and ([directory_2] = '1' or [directory_2] = '3')	y
attitudes_9_intensive_care	Being infected and requiring intensive hospital care (mechanical ventilation or a breathing machine)	1, To a Great Extent | 2, Somewhat | 3, Very Little | 4, Not at All | 5, I don't know/I prefer not to answer/Not applicable	[directory_1] = '1' and ([directory_2] = '1' or [directory_2] = '3')	y
attitudes_9_death	Being infected and dying	1, To a Great Extent | 2, Somewhat | 3, Very Little | 4, Not at All | 5, I don't know/I prefer not to answer/Not applicable	[directory_1] = '1' and ([directory_2] = '1' or [directory_2] = '3')	y
attitudes_9_hospital_other	Requiring hospitalization for other non-COVID-19 related health complications	1, To a Great Extent | 2, Somewhat | 3, Very Little | 4, Not at All | 5, I don't know/I prefer not to answer/Not applicable	[directory_1] = '1' and ([directory_2] = '1' or [directory_2] = '3')	y
attitudes_9_not_soc_dis	Not being able to social distance because of required assistance due to Parkinson's	1, To a Great Extent | 2, Somewhat | 3, Very Little | 4, Not at All | 5, I don't know/I prefer not to answer/Not applicable	[directory_1] = '1' and ([directory_2] = '1' or [directory_2] = '3')	y
attitudes_9_pd_meds	Running out of Parkinson's medications	1, To a Great Extent | 2, Somewhat | 3, Very Little | 4, Not at All | 5, I don't	[directory_1] = '1' and ([directory_2] = '1' or [directory_2] = '3')	y

		know/I prefer not to answer/Not applicable		
attitudes_9_money	Not having enough money for living expenses	1, To a Great Extent | 2, Somewhat | 3, Very Little | 4, Not at All | 5, I don't know/I prefer not to answer/Not applicable		y
attitudes_9_time	How long it will take for things to go back to normal	1, To a Great Extent | 2, Somewhat | 3, Very Little | 4, Not at All | 5, I don't know/I prefer not to answer/Not applicable	[directory_1] = '1' and ([directory_2] = '1' or [directory_2] = '3')	y
attitudes_10_nervous	Feeling nervous, anxious, or on edge	1, Nearly every day | 2, More than half the days | 3, Several days | 4, Not at all	[directory_1] = '1' and ([directory_2] = '1' or [directory_2] = '3')	y
attitudes_10_worry	Not being able to stop or control worrying	1, Nearly every day | 2, More than half the days | 3, Several days | 4, Not at all	[directory_1] = '1' and ([directory_2] = '1' or [directory_2] = '3')	y
attitudes_10_depressed	Feeling down, depressed, or hopeless	1, Nearly every day | 2, More than half the days | 3, Several days | 4, Not at all	[directory_1] = '1' and ([directory_2] = '1' or [directory_2] = '3')	y
attitudes_10_loss_interest	Little interest or pleasure in doing things	1, Nearly every day | 2, More than half the days | 3, Several days | 4, Not at all	[directory_1] = '1' and ([directory_2] = '1' or [directory_2] = '3')	y
attitudes_10_sleep	Had sleep interruptions or disturbances	1, Nearly every day | 2, More than half the days | 3, Several days | 4, Not at all	[directory_1] = '1' and ([directory_2] = '1' or [directory_2] = '3')	y
attitudes_10_isolated	Feeling isolated, left out or alone	1, Nearly every day | 2, More than half the days | 3, Several days | 4, Not at all	[directory_1] = '1' and ([directory_2] = '1' or [directory_2] = '3')	y
attitudes_10_1_nervous	In what ways do you feel that disruptions caused by the coronavirus outbreak have contributed to feeling nervous, anxious, or on edge?		[attitudes_10_nervous] = '1' or [attitudes_10_nervous] = '2'	
attitudes_10_1_worry	In what ways do you feel that disruptions caused by the coronavirus outbreak have contributed to not being able to stop or control worrying?		[attitudes_10_worry] = '1' or [attitudes_10_worry] = '2'	
attitudes_10_1_depressed	In what ways do you feel that disruptions caused by the coronavirus outbreak have contributed to feeling down, depressed, or hopeless?		[attitudes_10_depressed] = '1' or [attitudes_10_depressed] = '2'	
attitudes_10_1_loss_interest	In what ways do you feel that disruptions caused by the coronavirus outbreak have contributed to little interest or pleasure in doing things?		[attitudes_10_loss_interest] = '1' or [attitudes_10_loss_interest] = '2'	
attitudes_10_1_sleep	In what ways do you feel that disruptions caused by the coronavirus outbreak have contributed to sleep interruptions or disturbances?		[attitudes_10_sleep] = '2' or [attitudes_10_sleep] = '1'	
attitudes_10_1_isolated	In what ways do you feel that disruptions caused by the coronavirus outbreak have contributed to feeling isolated, left out or alone?		[attitudes_10_isolated] = '1' or [attitudes_10_isolated] = '2'	

attitudes_11	During the last six weeks, have you noticed a change in your Parkinson's disease symptoms?	1, Yes, major negative change | 2, Yes, minor negative change | 3, Yes, major positive change | 4, Yes, minor positive change | 5, No, Parkinson's disease symptoms have remained about the same | 6, Not sure	[directory_1] = '1' and ([directory_2] = '1' or [directory_2] = '3')	y
attitudes_11_1	In what ways do you feel that the disruptions caused by the coronavirus outbreak have contributed to negative changes in your Parkinson's disease symptoms?		[attitudes_11] = '1'	y
attitudes_12	During the last six weeks, have you noticed a change in your relationships with members of your household?	1, Yes, major negative change | 2, Yes, minor negative change | 3, Yes, major positive change | 4, Yes, minor positive change | 5, No, relationships with members of my household have remained about the same | 6, Not sure | 7, I live alone | 8, Not sure	[directory_1] = '1' and ([directory_2] = '1' or [directory_2] = '3')	y
attitudes_12_1	In what ways do you feel that the disruptions caused by the coronavirus has contributed to negative changes in your relationships with members of your household?		[attitudes_12] = '1'	y
attitudes_13	Are there things that you don't understand or that confuse you about the coronavirus?	1, Yes | 2, No | 3, Not sure	[directory_1] = '1' and ([directory_2] = '1' or [directory_2] = '3')	y
attitudes_13_1	What is it that you don't understand, or what confuses you about the coronavirus?		[attitudes_13] = '1'	y
attitudes_14	What additional information or resources would be most helpful to you at this time?		[directory_1] = '1' and ([directory_2] = '1' or [directory_2] = '3')	
practices_16_travel	Canceled or postponed air travel for work or pleasure	1, Yes | 2, No | 3, Not applicable	[directory_1] = '1' and ([directory_2] = '1' or [directory_2] = '3')	y
practices_16_activities	Canceled or postponed personal or social activities	1, Yes | 2, No | 3, Not applicable	[directory_1] = '1' and ([directory_2] = '1' or [directory_2] = '3')	y
practices_16_doctor	Visited a doctor	1, Yes | 2, No | 3, Not applicable	[directory_1] = '1' and ([directory_2] = '1' or [directory_2] = '3')	y
practices_16_cancelled_apt	Canceled a doctor's appointment	1, Yes | 2, No | 3, Not applicable	[directory_1] = '1' and ([directory_2] = '1' or [directory_2] = '3')	y

practices_16_food	Stockpiled food or water	1, Yes | 2, No | 3, Not applicable	[directory_1] = '1' and ([directory_2] = '1' or [directory_2] = '3')	y
practices_16_avoid_people	Avoided contact with people who could be high-risk	1, Yes | 2, No | 3, Not applicable	[directory_1] = '1' and ([directory_2] = '1' or [directory_2] = '3')	y
practices_16_avoid_places	Avoided public spaces, gatherings, or crowds	1, Yes | 2, No | 3, Not applicable	[directory_1] = '1' and ([directory_2] = '1' or [directory_2] = '3')	y
practices_16_avoid_restaurant	Avoided eating at restaurants	1, Yes | 2, No | 3, Not applicable	[directory_1] = '1' and ([directory_2] = '1' or [directory_2] = '3')	y
practices_16_pray	Prayed	1, Yes | 2, No | 3, Not applicable	[directory_1] = '1' and ([directory_2] = '1' or [directory_2] = '3')	y
practices_16_pd_meds	Obtained a three-month supply of Parkinson's prescription medications	1, Yes | 2, No | 3, Not applicable	[directory_1] = '1' and ([directory_2] = '1' or [directory_2] = '3')	y
practices_16_other_meds	Obtained a three-month supply of other essential prescription medications	1, Yes | 2, No | 3, Not applicable	[directory_1] = '1' and ([directory_2] = '1' or [directory_2] = '3')	y
practices_16_wfh	Worked or studied at home	1, Yes | 2, No | 3, Not applicable	[directory_1] = '1' and ([directory_2] = '1' or [directory_2] = '3')	y
practices_16_mask	Worn a mask or other face covering	1, Yes | 2, No | 3, Not applicable	[directory_1] = '1' and ([directory_2] = '1' or [directory_2] = '3')	y
practices_16_1	<div class="rich-text-field-label"><p>Were you unable to get any of the following items as a result of the coronavirus outbreak? Please select all that apply.</p></div>	1, Prescription medications for Parkinson's disease | 2, Other prescription medications | 3, Groceries | 4, Other necessary items	[practices_16_pd_meds] = '2' or [practices_16_other_meds] = '2' or [practices_16_food] = '2'	
practices_17	Has anything prevented you from following the CDC and government social distancing and	1, Yes | 2, No	[directory_1] = '1' and ([directory_2] = '1' or [directory_2] = '3')	y

	coronavirus health recommendations?			
practices_17_1	What has prevented you from following the CDC and government social distancing and coronavirus health recommendations?		[practices_17] = '1'	y
practices_18	Prior to the coronavirus outbreak, approximately how many hours per week did you exercise?	1, I did not exercise | 2, <1 hour per week | 3, 1-3 hours per week | 4, 4-6 hours per week | 5, 7-9 hours per week | 6, 10+ hours per week	[directory_1] = '1' and ([directory_2] = '1' or [directory_2] = '3')	y
practices_19	During the last six weeks, approximately how many hours per week have you exercised?	1, I did not exercise | 2, < 1 hour per week | 3, 1-3 hours per week | 4, 4-6 hours per week | 5, 7-9 hours per week | 6, 10+ hours per week	[directory_1] = '1' and ([directory_2] = '1' or [directory_2] = '3')	y
practices_20	Prior to the coronavirus outbreak, approximately how many hours per week did you participate in activities outside of your residence? Activities can include exercise or wellness classes, support groups, recreational classes, religious services, educational events, etc.	1, I did not leave my residence | 2, < 1 hour per week | 3, 1-5 hours per week | 4, 6-10 hours per week | 5, 11-15 hours per week | 6, 16+ hours per week	[directory_1] = '1' and ([directory_2] = '1' or [directory_2] = '3')	y
practices_21	During the last six weeks, approximately how many hours per week did you participate in activities outside of your residence? Activities can include exercise or wellness classes, support groups, recreational classes, religious services, educational events, etc.	1, I did not leave my residence | 2, < 1 hour per week | 3, 1-5 hours per week | 4, 6-10 hours per week | 5, 11-15 hours per week | 6, 16+ hours per week	[directory_1] = '1' and ([directory_2] = '1' or [directory_2] = '3')	y
practices_22	Compared to the months before the outbreak began, how has the frequency of your communication with close friends and family changed?	1, I communicate with them more often than before | 2, I communicate with them about the same as before | 3, I communicate with them less often than before	[directory_1] = '1' and ([directory_2] = '1' or [directory_2] = '3')	y
practices_23	Who is providing you with social support during the coronavirus outbreak? Please select all that apply.	1, Someone in my household or residence | 2, Friend or family who comes to my place | 3, Friend or family who I talk to on the phone (or video	[directory_1] = '1' and ([directory_2] = '1' or [directory_2] = '3')	y

		chat) | 4, Other | 5, I do not have support		
practices_23_1	Who else is providing you with social support during the outbreak?		[practices_23(4)] = '1'	y
telehealth_24	Were any of the activities you would participate in previous to the coronavirus outbreak transitioned to a virtual or online format? Activities can include exercise or wellness classes, support groups, recreational classes, religious services, educational events, etc.	1, All | 2, Some | 3, Few | 4, None	[directory_1] = '1' and ([directory_2] = '1' or [directory_2] = '3')	y
telehealth_24_1	Have you participated in those activities in a virtual or online format?	1, All | 2, Some | 3, Few | 4, None	[telehealth_24] = '1' or [telehealth_24] = '2' or [telehealth_24] = '3'	y
telehealth_24_2	During the last six weeks, in what activities have you participated in a virtual or online format? Please select all that apply.	1, Exercise or wellness classes | 2, Support groups | 3, Recreational classes (art, dance, drama, etc.) | 4, Religious services | 5, Educational events | 6, Other	[telehealth_24_1] = '1' or [telehealth_24_1] = '2' or [telehealth_24_1] = '3'	y
telehealth_24_2_1	In what other activities have you participated in a virtual or online format?		[telehealth_24_2(6)] = '1'	y
telehealth_25	How long has it been since you last visited (in person appointment) the doctor or medical provider you see most often for treatment related to your Parkinson's?	1, Less than one month | 2, 1 to 3 months | 3, 4 to 6 months | 4, 7 to 12 months | 5, More than 12 months	[directory_1] = '1' and ([directory_2] = '1' or [directory_2] = '3')	y
telehealth_26_doctor	Doctor or medical provider	2, 1 appointment | 3, 2 appointments | 4, 3 to 5 appointments | 5, 6 or more appointments | 1, I did not receive this care via telehealth or virtual appointments	[directory_1] = '1' and ([directory_2] = '1' or [directory_2] = '3')	y
telehealth_26_pt	Physical therapist	2, 1 appointment | 3, 2 appointments | 4, 3 to 5 appointments | 5, 6 or more appointments | 1, I did not receive this care via telehealth or virtual appointments	[directory_1] = '1' and ([directory_2] = '1' or [directory_2] = '3')	y

telehealth_26_ot	Occupational therapist	2, 1 appointment | 3, 2 appointments | 4, 3 to 5 appointments | 5, 6 or more appointments | 1, I did not receive this care via telehealth or virtual appointments	[directory_1] = '1' and ([directory_2] = '1' or [directory_2] = '3')	y
telehealth_26_slp	Speech and language pathologist	2, 1 appointment | 3, 2 appointments | 4, 3 to 5 appointments | 5, 6 or more appointments | 1, I did not receive this care via telehealth or virtual appointments	[directory_1] = '1' and ([directory_2] = '1' or [directory_2] = '3')	y
telehealth_26_mental	Mental health therapist	2, 1 appointment | 3, 2 appointments | 4, 3 to 5 appointments | 5, 6 or more appointments | 1, I did not receive this care via telehealth or virtual appointments	[directory_1] = '1' and ([directory_2] = '1' or [directory_2] = '3')	y
telehealth_26_2_doctor	How was this telehealth or virtual appointment with a doctor of medical provider conducted?	1, Over a video sharing app on your smart phone | 2, Over video on a computer | 3, Over the phone with audio only | 4, Other	[telehealth_26_doctor] = '2' or [telehealth_26_doctor] = '3' or [telehealth_26_doctor] = '4' or [telehealth_26_doctor] = '5'	y
telehealth_26_2_pt	How was this telehealth or virtual appointment with a physical therapist conducted?	1, Over a video sharing app on your smart phone | 2, Over video on a computer | 3, Over the phone with audio only | 4, Other	[telehealth_26_pt] = '2' or [telehealth_26_pt] = '3' or [telehealth_26_pt] = '4' or [telehealth_26_pt] = '5'	y
telehealth_26_2_ot	How was this telehealth or virtual appointment with an occupational therapist conducted?	1, Over a video sharing app on your smart phone | 2, Over video on a computer | 3, Over the phone with audio only | 4, Other	[telehealth_26_ot] = '2' or [telehealth_26_ot] = '3' or [telehealth_26_ot] = '4' or [telehealth_26_ot] = '5'	y
telehealth_26_2_slp	How was this telehealth or virtual appointment with a speech and language pathologist conducted?	1, Over a video sharing app on your smart phone | 2, Over video on a computer | 3, Over the phone with audio only | 4, Other	[telehealth_26_slp] = '2' or [telehealth_26_slp] = '3' or [telehealth_26_slp] = '4' or [telehealth_26_slp] = '5'	y
telehealth_26_2_mental	How was this telehealth or virtual appointment with a mental health therapist conducted?	1, Over a video sharing app on your smart phone | 2, Over video on a computer | 3, Over the phone with audio only | 4, Other	[telehealth_26_mental] = '2' or [telehealth_26_mental] = '3' or [telehealth_26_mental] = '4' or [telehealth_26_mental] = '5'	y
telehealth_26_3_doctor	Were any of your telehealth or virtual appointments related to	1, Routine visit for Parkinson's disease | 2, Urgent visit for Parkinson's disease	[telehealth_26_doctor] = '2' or [telehealth_26_doctor] =	y

	any of the following medical issues?	| 3, Corona virus symptoms, either to diagnosis or to rule out | 4, Other urgent health situation | 5, None of the above	'3' or [telehealth_26_doctor] = '4' or [telehealth_26_doctor] = '5'	
telehealth_26_4	Were you provided support or instructions on how to use telehealth or virtual services for this appointment?	1, I was provided support (by medical staff or someone familiar with technology) | 2, I was provided instructions | 3, I was provided both support and instructions | 4, I was not provided support or instructions	[telehealth_26_doctor] = '2' or [telehealth_26_doctor] = '3' or [telehealth_26_doctor] = '4' or [telehealth_26_doctor] = '5' or [telehealth_26_pt] = '2' or [telehealth_26_pt] = '3' or [telehealth_26_pt] = '4' or [telehealth_26_pt] = '5' or [telehealth_26_ot] = '2' or [telehealth_26_ot] = '3' or [telehealth_26_ot] = '4' or [telehealth_26_ot] = '5' or [telehealth_26_slp] = '2' or [telehealth_26_slp] = '3' or [telehealth_26_slp] = '4' or [telehealth_26_slp] = '5' or [telehealth_26_mental] = '2' or [telehealth_26_mental] = '3' or [telehealth_26_mental] = '4' or [telehealth_26_mental] = '5'	y
telehealth_26_5	Has a care partner, friend or family member been helping you with your telehealth or virtual medical appointments? Select all that apply.	1, Yes, holding the phone or device for me | 2, Yes, helping me with the technology (logging in, setting up audio or video, etc.) | 3, Yes, communicating with my doctor for or with me | 4, Yes, taking notes while my doctor is speaking | 5, Yes, other | 6, No, I am attending telehealth or virtual medical appointments on my own with no assistance	[telehealth_26_doctor] = '2' or [telehealth_26_doctor] = '3' or [telehealth_26_doctor] = '4' or [telehealth_26_doctor] = '5' or [telehealth_26_pt] = '2' or [telehealth_26_pt] = '3' or [telehealth_26_pt] = '4' or [telehealth_26_pt] = '5' or [telehealth_26_ot] = '2' or [telehealth_26_ot] = '3' or [telehealth_26_ot] = '4' or [telehealth_26_ot] = '5' or [telehealth_26_slp] = '2' or	y

			[telehealth_26_slp] = '3' or [telehealth_26_slp] = '4' or [telehealth_26_slp] = '5' or [telehealth_26_mental] = '2' or [telehealth_26_mental] = '3' or [telehealth_26_mental] = '4' or [telehealth_26_mental] = '5'	
telehealth_27	Prior to the coronavirus outbreak, had you used telehealth or virtual medical appointments before?	1, Yes | 2, No | 3, Not sure	[directory_1] = '1' and ([directory_2] = '1' or [directory_2] = '3')	y
telehealth_27_1	Were you provided with telehealth or virtual medical appointment options for the appointments you were required to cancel due to the coronavirus outbreak?	1, Yes, and I participated in the telehealth or virtual medical appointment | 2, Yes, but I did not participate in the telehealth or virtual medical appointment | 3, No, I was not provided telehealth or virtual medical appointment options | 4, Not sure	[practices_16_cancelled_apt] = '1'	y
telehealth_28_doctor	Doctor or medical provider	1, More satisfied | 2, Equally satisfied | 3, Less satisfied | 4, I'm not sure | 5, I did not receive this care via telehealth or virtual appointments	[directory_1] = '1' and ([directory_2] = '1' or [directory_2] = '3')	y
telehealth_28_pt	Physical therapist	1, More satisfied | 2, Equally satisfied | 3, Less satisfied | 4, I'm not sure | 5, I did not receive this care via telehealth or virtual appointments	[directory_1] = '1' and ([directory_2] = '1' or [directory_2] = '3')	y
telehealth_28_ot	Occupational therapist	1, More satisfied | 2, Equally satisfied | 3, Less satisfied | 4, I'm not sure | 5, I did not receive this care via telehealth or virtual appointments	[directory_1] = '1' and ([directory_2] = '1' or [directory_2] = '3')	y
telehealth_28_slp	Speech and language pathologist	1, More satisfied | 2, Equally satisfied | 3, Less satisfied | 4, I'm not sure | 5, I did not receive this care via telehealth or virtual appointments	[directory_1] = '1' and ([directory_2] = '1' or [directory_2] = '3')	y
telehealth_28_mental	Mental health therapist	1, More satisfied | 2, Equally satisfied | 3, Less satisfied | 4, I'm not sure | 5, I did not receive this care via telehealth or virtual appointments	[directory_1] = '1' and ([directory_2] = '1' or [directory_2] = '3')	y

telehealth_29	How frequently would you prefer to continue telehealth or virtual medical appointments after the coronavirus outbreak has ended?	1, Always, I prefer to only use telehealth or virtual medical appointments moving forward | 2, Sometimes, I prefer to use telehealth or virtual medical appointments as a replacement to some of my in-person appointments moving forward | 3, Rarely, I prefer to have in-person appointments and only use telehealth or virtual medical appointments as a replacement when absolutely necessary | 4, Never, I prefer to return to in-person appointments only	[directory_1] = '1' and ([directory_2] = '1' or [directory_2] = '3') and ([telehealth_26_doctor] = '2' or [telehealth_26_doctor] = '3' or [telehealth_26_doctor] = '4' or [telehealth_26_doctor] = '5' or [telehealth_26_pt] = '2' or [telehealth_26_pt] = '3' or [telehealth_26_pt] = '4' or [telehealth_26_pt] = '5' or [telehealth_26_ot] = '2' or [telehealth_26_ot] = '3' or [telehealth_26_ot] = '4' or [telehealth_26_ot] = '5' or [telehealth_26_slp] = '2' or [telehealth_26_slp] = '3' or [telehealth_26_slp] = '4' or [telehealth_26_slp] = '5' or [telehealth_26_mental] = '2' or [telehealth_26_mental] = '3' or [telehealth_26_mental] = '4' or [telehealth_26_mental] = '5')	y
telehealth_29_1	For what reasons do you prefer in person medical appointments to telehealth or virtual medical appointments?	1, In person medical appointment are more convenient than telehealth or virtual appointments | 2, In person appointments have higher quality of care than telehealth or virtual appointments | 3, There are too many technical challenges with telehealth or virtual appointments | 4, Other	[telehealth_29] = '3' or [telehealth_29] = '4'	y
telehealth_29_1_1	For what other reason do you prefer in person medical appointments to telehealth or virtual medical appointments?		[telehealth_29_1] = '4'	y
demographics_a	In what year were you born?	1915, 1915 | 1916, 1916 | 1917, 1917 | 1918, 1918 | 1919, 1919 | 1920, 1920 | 1921, 1921 | 1922, 1922 | 1923, 1923 | 1924, 1924 | 1925, 1925 | 1926, 1926 | 1927, 1927 | 1928,	[directory_1] = '1' and ([directory_2] = '1' or [directory_2] = '3')	y

		1928 | 1929, 1929 | 1930, 1930 | 1931, 1931 | 1932, 1932 | 1933, 1933 | 1934, 1934 | 1935, 1935 | 1936, 1936 | 1937, 1937 | 1938, 1938 | 1939, 1939 | 1940, 1940 | 1941, 1941 | 1942, 1942 | 1943, 1943 | 1944, 1944 | 1945, 1945 | 1946, 1946 | 1947, 1947 | 1948, 1948 | 1949, 1949 | 1950, 1950 | 1951, 1951 | 1952, 1952 | 1953, 1953 | 1954, 1954 | 1955, 1955 | 1956, 1956 | 1957, 1957 | 1958, 1958 | 1959, 1959 | 1960, 1960 | 1961, 1961 | 1962, 1962 | 1963, 1963 | 1964, 1964 | 1965, 1965 | 1966, 1966 | 1967, 1967 | 1968, 1968 | 1969, 1969 | 1970, 1970 | 1971, 1971 | 1972, 1972 | 1973, 1973 | 1974, 1974 | 1975, 1975 | 1976, 1976 | 1977, 1977 | 1978, 1978 | 1979, 1979 | 1980, 1980 | 1981, 1981 | 1982, 1982 | 1983, 1983 | 1984, 1984 | 1985, 1985 | 1986, 1986 | 1987, 1987 | 1988, 1988 | 1989, 1989 | 1990, 1990 | 1991, 1991 | 1992, 1992 | 1993, 1993 | 1994, 1994 | 1995, 1995 | 1996, 1996 | 1997, 1997 | 1998, 1998 | 1999, 1999 | 2000, 2000		
demographics_b	In what year were you diagnosed Parkinson's disease?	1, Prior to 1950 | 1950, 1950 | 1951, 1951 | 1952, 1952 | 1953, 1953 | 1954, 1954 | 1955, 1955 | 1956, 1956 | 1957, 1957 | 1958, 1958 | 1959, 1959 | 1960, 1960 | 1961, 1961 | 1962, 1962 | 1963, 1963 | 1964, 1964 | 1965, 1965 | 1966, 1966 | 1967, 1967 | 1968, 1968 | 1969, 1969 | 1970, 1970 | 1971, 1971 | 1972, 1972 | 1973, 1973 | 1974, 1974 | 1975, 1975 | 1976, 1976 | 1977, 1977 | 1978, 1978 | 1979, 1979 | 1980, 1980	[directory_1] = '1' and ([directory_2] = '1' or [directory_2] = '3')	y

		| 1981, 1981 | 1982, 1982 | 1983, 1983 | 1984, 1984 | 1985, 1985 | 1986, 1986 | 1987, 1987 | 1988, 1988 | 1989, 1989 | 1990, 1990 | 1991, 1991 | 1992, 1992 | 1993, 1993 | 1994, 1994 | 1995, 1995 | 1996, 1996 | 1997, 1997 | 1998, 1998 | 1999, 1999 | 2000, 2000 | 2001, 2001 | 2002, 2002 | 2003, 2003 | 2004, 2004 | 2005, 2005 | 2006, 2006 | 2007, 2007 | 2008, 2008 | 2009, 2009 | 2010, 2010 | 2011, 2011 | 2012, 2012 | 2013, 2013 | 2014, 2014 | 2015, 2015 | 2016, 2016 | 2017, 2017 | 2018, 2018 | 2019, 2019 | 2020, 2020		
demographics_c	What medications do you currently take for your Parkinson's disease? Please select all that apply.	1, L-Dopa (Medications include: Sinemet, Sinemet CR, Parcopa, Stalevo, Rytary, Duopa) | 2, Dopamine Agonists (Medications include: Apokyn, Parlodel, Mirapex, Mirapex ER, Requip, Requip XLTM, Neupro) | 3, COMT Inhibitors (Medications include: Comtan, Tasmar) | 4, MAO-B Inhibitors (Azilect, Eldepryl, Zelapar, Xadago) | 5, Anticholinergics (Medications include: Cogentin, Artane) | 6, Other (Medications include: Gocovri, Northera, Nuplazid, Exelon) | 7, I do not currently take medications for my Parkinson's disease	[directory_1] = '1' and ([directory_2] = '1' or [directory_2] = '3')	y
demographics_d	Do you have a care partner or caregiver?	1, Yes | 2, No	[directory_1] = '1' and ([directory_2] = '1' or [directory_2] = '3')	y
demographics_e	What is your marital status?	1, Single (never married) | 2, Married or domestic partnership | 3, Separated | 4, Widowed | 5, Divorced	[directory_1] = '1' and ([directory_2] = '1' or [directory_2] = '3')	y
demographics_f	Please select the gender you identify with:	1, Male | 2, Female | 3, Other | 4, Prefer to not answer	[directory_1] = '1' and ([directory_2] = '1' or [directory_2] = '3')	y

demographics_g	Please select the categories that describe you: Please select all that apply.	1, American Indian or Alaska Native | 2, Asian | 3, Black/African American | 4, Hispanic/Latino | 5, Native Hawaiian Pacific Islander | 6, White | 7, Mixed | 8, Some other race, ethnicity or origin ____ | 9, Prefer to not answer	[directory_1] = '1' and ([directory_2] = '1' or [directory_2] = '3')	y
demographics_h	What is your current employment status?	1, Employed full time | 2, Employed part time | 3, Self-employed | 4, Unemployed | 5, Retired | 6, Disability or unable to work	[directory_1] = '1' and ([directory_2] = '1' or [directory_2] = '3')	y
demographics_i	What is your highest level of education?	1, Less than a high school diploma | 2, High school degree or equivalent (e.g. GED) | 3, Some college, no degree | 4, Trade/technical school (vocational training) | 5, Associate degree (e.g. AA, AS) | 6, Bachelor's degree (e.g. BA, BS) | 7, Master's degree (e.g. MA, MS, MEd) | 8, Professional degree (e.g. MD, DDS, DVM) | 9, Doctorate (e.g. PhD, EdD)	[directory_1] = '1' and ([directory_2] = '1' or [directory_2] = '3')	y
demographics_j	What is your current yearly household income? include income from Social Security, investment, etc.)	1, Less than $25,000 | 2, $25,000 to $34,999 | 3, $35,000 to $49,999 | 4, $50,000 to $74,999 | 5, $75,000 to $99,999 | 6, $100,000 to $149,999 | 7, $150,000 to $199,999 | 8, $200,000 or more | 9, Prefer not to say	[directory_1] = '1' and ([directory_2] = '1' or [directory_2] = '3')	y
demographics_k	In what country do you currently live?	1, United States | 2, Afghanistan | 3, Albania | 4, Algeria | 5, Andorra | 6, Angola | 7, Antigua and Barbuda | 8, Argentina | 9, Armenia | 10, Australia | 11, Austria | 12, Azerbaijan | 13, The Bahamas | 14, Bahrain | 15, Bangladesh | 16, Barbados | 17, Belarus | 18, Belgium | 19, Belize | 20, Benin | 21, Bhutan | 22, Bolivia | 23, Bosnia and Herzegovina | 24, Botswana | 25, Brazil | 26, Brunei | 27, Bulgaria | 28, Burkina Faso | 29, Burundi | 30, Cabo Verde | 31,	[directory_1] = '1' and ([directory_2] = '1' or [directory_2] = '3')	y

		Cambodia | 32, Cameroon | 33, Canada | 34, Central African Republic | 35, Chad | 36, Chile | 37, China | 38, Colombia | 39, Comoros | 40, Congo Democratic Republic of the | 41, Congo Republic of the | 42, Costa Rica | 43, Côte d'Ivoire | 44, Croatia | 45, Cuba | 46, Cyprus | 47, Czech Republic | 48, Denmark | 49, Djibouti | 50, Dominica | 51, Dominican Republic | 52, East Timor (Timor-Leste) | 53, Ecuador | 54, Egypt | 55, El Salvador | 56, Equatorial Guinea | 57, Eritrea | 58, Estonia | 59, Eswatini | 60, Ethiopia | 61, Fiji | 62, Finland | 63, France | 64, Gabon | 65, The Gambia | 66, Georgia | 67, Germany | 68, Ghana | 69, Greece | 70, Grenada | 71, Guatemala | 72, Guinea | 73, Guinea-Bissau | 74, Guyana | 75, Haiti | 76, Honduras | 77, Hungary | 78, Iceland | 79, India | 80, Indonesia | 81, Iran | 82, Iraq | 83, Ireland | 84, Israel | 85, Italy | 86, Jamaica | 87, Japan | 88, Jordan | 89, Kazakhstan | 90, Kenya | 91, Kiribati | 92, Korea North | 93, Korea South | 94, Kosovo | 95, Kuwait | 96, Kyrgyzstan | 97, Laos | 98, Latvia | 99, Lebanon | 100, Lesotho | 101, Liberia | 102, Libya | 103, Liechtenstein | 104, Lithuania | 105, Luxembourg | 106, Madagascar | 107, Malawi | 108, Malaysia | 109, Maldives | 110, Mali | 111, Malta | 112, Marshall Islands | 113, Mauritania | 114, Mauritius | 115, Mexico | Micronesia, Federated States of | 116, Moldova | 117, Monaco | 118, Mongolia | 119, Montenegro | 120, Morocco | 121, Mozambique | 122,		

		Myanmar (Burma) | 123, Namibia | 124, Nauru | 125, Nepal | 126, Netherlands | 127, New Zealand | 128, Nicaragua | 129, Niger | 130, Nigeria | 131, North Macedonia | 132, Norway | 133, Oman | 134, Pakistan | 135, Palau | 136, Panama | 137, Papua New Guinea | 138, Paraguay | 139, Peru | 140, Philippines | 141, Poland | 142, Portugal | 143, Qatar | 144, Romania | 145, Russia | 146, Rwanda | 147, Saint Kitts and Nevis | 148, Saint Lucia | 149, Saint Vincent and the Grenadines | 150, Samoa | 151, San Marino | 152, Sao Tome and Principe | 153, Saudi Arabia | 154, Senegal | 155, Serbia | 156, Seychelles | 157, Sierra Leone | 158, Singapore | 159, Slovakia | 160, Slovenia | 161, Solomon Islands | 162, Somalia | 163, South Africa | 164, Spain | 165, Sri Lanka | 166, Sudan | 167, Sudan South | 168, Suriname | 169, Sweden | 170, Switzerland | 171, Syria | 172, Taiwan | 173, Tajikistan | 174, Tanzania | 175, Thailand | 176, Togo | 177, Tonga | 178, Trinidad and Tobago | 179, Tunisia | 180, Turkey | 181, Turkmenistan | 182, Tuvalu | 183, Uganda | 184, Ukraine | 185, United Arab Emirates | 186, United Kingdom | 187, Uruguay | 188, Uzbekistan | 189, Vanuatu | 190, Vatican City | 191, Venezuela | 192, Vietnam | 193, Yemen | 194, Zambia | 195, Zimbabwe		
demographics_m	In what state do you currently live?	1, Alabama | 2, Alaska | 3, Arizona | 4, Arkansas | 5, California | 6, Colorado | 7, Connecticut | 8, Delaware | 9, Florida | 10, Georgia | 11, Hawaii | 12, Idaho | 13, Illinois | 14, Indiana | 15, Iowa | 16, Kansas | 17, Kentucky | 18,	[directory_1] = '1' and ([directory_2] = '1' or [directory_2] = '3') and [demographics_k] = '1'	y

		Louisiana | 19, Maine | 20, Maryland | 21, Massachusetts | 22, Michigan | 23, Minnesota | 24, Mississippi | 25, Missouri | 26, Montana | 27, Nebraska | 28, Nevada | 29, New Hampshire | 30, New Jersey | 31, New Mexico | 32, New York | 33, North Carolina | 34, North Dakota | 35, Ohio | 36, Oklahoma | 37, Oregon | 38, Pennsylvania | 39, Rhode Island | 40, South Carolina | 41, South Dakota | 42, Tennessee | 43, Texas | 44, Utah | 45, Vermont | 46, Virginia | 47, Washington | 48, West Virginia | 49, Wisconsin | 50, Wyoming		
demographics_l	In what city do you currently live?		[directory_1] = '1' and ([directory_2] = '1' or [directory_2] = '3')	y
demographics_n	Because location can be a risk factor for the coronavirus, please also provide your current zip code.		[directory_1] = '1' and ([directory_2] = '1' or [directory_2] = '3')	
demographics_o	Did anyone help you complete this survey today?	1, Yes, my care partner | 2, Yes, a family member | 3, Yes, a friend | 4, No, I completed the survey on my own	[directory_1] = '1' and ([directory_2] = '1' or [directory_2] = '3')	y
